# A Trihelix DNA Binding Protein Counterbalances Hypoxia-Responsive Transcriptional Activation in Arabidopsis

**DOI:** 10.1371/journal.pbio.1001950

**Published:** 2014-09-16

**Authors:** Beatrice Giuntoli, Seung Cho Lee, Francesco Licausi, Monika Kosmacz, Teruko Oosumi, Joost T. van Dongen, Julia Bailey-Serres, Pierdomenico Perata

**Affiliations:** 1 Institute of Life Sciences, Scuola Superiore Sant'Anna, Pisa, Italy; 2 Center for Plant Cell Biology, University of California, Riverside, California, United States of America; 3 Max Planck Institute for Molecular Plant Physiology, Potsdam-Golm, Germany; 4 Institute of Biology, RWTH Aachen University, Aachen, Germany; University of California, Davis, United States of America

## Abstract

Transcriptional activation in response to hypoxia in plants is orchestrated by ethylene-responsive factor group VII (ERF-VII) transcription factors, which are stable during hypoxia but destabilized during normoxia through their targeting to the N-end rule pathway of selective proteolysis. Whereas the conditionally expressed ERF-VII genes enable effective flooding survival strategies in rice, the constitutive accumulation of N-end-rule–insensitive versions of the *Arabidopsis thaliana* ERF-VII factor RAP2.12 is maladaptive. This suggests that transcriptional activation under hypoxia that leads to anaerobic metabolism may need to be fine-tuned. However, it is presently unknown whether a counterbalance of RAP2.12 exists. Genome-wide transcriptome analyses identified an uncharacterized trihelix transcription factor gene, which we named *HYPOXIA RESPONSE ATTENUATOR1* (*HRA1*), as highly up-regulated by hypoxia. HRA1 counteracts the induction of core low oxygen-responsive genes and transcriptional activation of hypoxia-responsive promoters by RAP2.12. By yeast-two-hybrid assays and chromatin immunoprecipitation we demonstrated that HRA1 interacts with the RAP2.12 protein but with only a few genomic DNA regions from hypoxia-regulated genes, indicating that HRA1 modulates RAP2.12 through protein–protein interaction. Comparison of the low oxygen response of tissues characterized by different levels of metabolic hypoxia (i.e., the shoot apical zone versus mature rosette leaves) revealed that the antagonistic interplay between RAP2.12 and HRA1 enables a flexible response to fluctuating hypoxia and is of importance to stress survival. In Arabidopsis, an effective low oxygen-sensing response requires RAP2.12 stabilization followed by HRA1 induction to modulate the extent of the anaerobic response by negative feedback regulation of RAP2.12. This mechanism is crucial for plant survival under suboptimal oxygenation conditions. The discovery of the feedback loop regulating the oxygen-sensing mechanism in plants opens new perspectives for breeding flood-resistant crops.

## Abstract 2

DNA binding protein controls plant transcription when oxygen is at a premium - During hypoxia, the plant transcription factor HRA1 counterbalances the upregulation of anaerobic gene expression triggered by a stabilized plant ethylene responsive factor.

## Author Summary

Respiratory metabolism in land plants requires oxygen availability to be able to generate ATP, which is essential for biosynthetic processes. Cellular hypoxia can be triggered as a consequence of environmental events (mainly floods), anatomical constraints (low tissue permeability to gases), or elevated cellular respiration, and it is unfavorable to growth due to the resultant decline in ATP. The adaptation of plants to fluctuating oxygen levels inside tissues requires the dynamic regulation of mechanisms that ensure cell viability and ultimately organism survival, but only a few molecular components of this homeostatic network are known. Direct hypoxia-sensing entails the posttranslational stabilization of a subgroup of plant ethylene-responsive factor (ERF) transcription factors, which coordinate the expression of hypoxia-inducible genes. Turnover of these ERFs is determined by an oxygen-dependent pathway of proteasomal degradation. Here, we demonstrate that the hypoxia-inducible transcription factor gene *HRA1* is transcriptionally activated upon ERF-VII RAP2.12 stabilization and encodes a trihelix DNA binding protein that functionally interacts with RAP2.12 to curtail its activity. In addition to its negative regulation of RAP2.12, HRA1 negatively regulates activation of its own promoter. This RAP2.12-HRA1 control unit allows plants to modulate the extent of the response to hypoxia, including anaerobic enzyme production, to levels that improve endurance of the stress. Our results emphasize the importance of a strategy that can counterbalance energy-inefficient survival responses.

## Introduction

In higher plants, respiratory metabolism requires molecular oxygen as the terminal electron acceptor to generate ATP. Limited oxygen availability (hypoxia) can occur in plant cells due to floods, frosts, and excessive respiration, requiring physiological acclimation to constraints in ATP availability for growth and development [1],[2]. The switch from aerobic respiration to anaerobic ethanolic fermentation, as a means to maintain substrate-level ATP production from available carbohydrates, is essential for plant survival in conditions of oxygen deprivation [3],[4]. For instance, mutants lacking pyruvate decarboxylase (PDC) or alcohol dehydrogenase (ADH), key enzymes in ethanolic fermentation, are less tolerant to hypoxia and soil waterlogging [4]–[6]. On the other hand, uncontrolled or constitutive fermentation is also detrimental to plant survival, due to rapid depletion of carbohydrate resources needed for basic cellular homeostasis [7]. The repression of catabolic metabolism is the basis of the quiescence survival strategy of the flash-flood–tolerant varieties of rice that have been recently adopted by many farmers in South and Southeastern Asia [8]. Molecular responses must be, thus, accurately balanced to meet plant requirements for survival under fluctuating oxygen conditions [9].

Low oxygen responses are coordinately regulated in plants by ethylene-responsive factor group VII (ERF-VII) transcription factors (TFs), primary activators of anaerobic gene expression [1]. In *Arabidopsis thaliana*, the presence of the ERF-VII factor RAP2.12 in the nucleus is inversely correlated to cellular oxygen levels, due to an oxygen-dependent branch of the N-end rule pathway of targeted protein degradation [7],[10]–[12]. It has been observed that constitutive accumulation of versions of RAP2.12 that are insensitive to the N-end rule degradation leads to a decreased submergence or hypoxia stress tolerance, whereas overexpression of the native RAP2.12 factor improves survival [7]. Therefore, excessive up-regulation of the stress-responsive genes appears to be detrimental, leading to the hypothesis that fine-tuning of transcription is a prerequisite for cellular homeostasis under hypoxia.

Among the genes that are induced by oxygen deficiency, those encoding known or putative TFs deserve special attention as candidate modulators of transcription under hypoxia. When hypoxia-responsive genes are compared across different plant species, a few TFs in addition to ERF-VIIs are consistently up-regulated by oxygen deprivation [13]. These include zinc finger, MADS, LOB domain proteins, and trihelix TF gene family members. The trihelix family, in particular, encompasses plant-specific TFs that have been so far linked to embryo development, trichome formation, seed shattering, and tolerance to biotic and abiotic stresses [14]. This study was aimed at the molecular and physiological characterization of a hypoxia-inducible trihelix TF gene (*At3g10040*), which we named *HRA1* (*HYPOXIA RESPONSE ATTENUATOR 1*). Here, we present evidence that *HRA1* encodes a transcriptional repressor that attenuates the anaerobic response induced by ERF-VIIs in a tissue-specific manner. We show that HRA1 imposes an additional level of negative regulation on RAP2.12, besides the ERF-VII's oxygen-dependent instability. RAP2.12 transcriptionally activates *HRA1*, which in turn binds RAP2.12 and restrains its function. Additionally, HRA1 interacts with its own promoter, limiting its activation by RAP2.12 through a negative feedback mechanism. Thus, transcriptional activation by RAP2.12 is controlled under normoxia by its N-end rule susceptibility and under oxygen deficiency by HRA1. The spatial and temporal regulation of both factors appears to be a key to modulation of transcriptional activity and survival of transient hypoxia.

## Results

### HRA1 Is a Low Oxygen-Inducible Nuclear Factor

**Figure 1:**
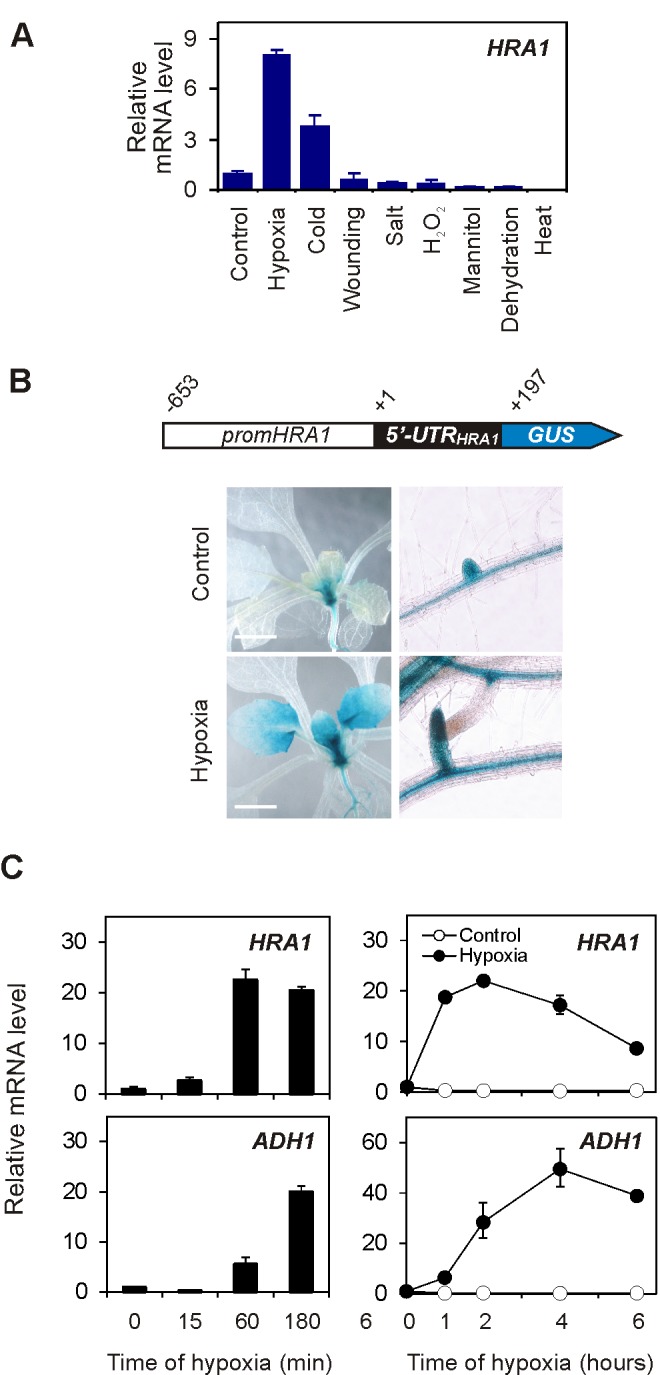
*HRA*1 is a low oxygen-inducible gene from Arabidopsis. (A) *HRA1* mRNA steady state levels in hypoxia-treated seedlings, in comparison with other abiotic stress treatments. Data are mean ± s.d. (*n* = 3). (B) Visualization of *HRA1* promoter activity by GUS-reporter staining. Nucleotide positions in the schematic are relative to *HRA1* transcription start site. Scale bar, 2 mm. (C) Transcript accumulation of *HRA1* and the hypoxic marker *ADH1* in seedlings, over an initial and more prolonged time course of sublethal hypoxia (upper and lower left diagrams). Data are mean ± s.d. (*n* = 3).

The *A. thaliana* Columbia-0 genome encodes 30 genes belonging to the plant-specific family of trihelix TFs [14]. A survey of public transcriptomic data showed that the gene *At3g10040* (*HRA1*) is the only trihelix family member up-regulated by oxygen deprivation (Figure S1). Trihelix TFs are induced by low oxygen in different species (Table S1 and Figure S2) and therefore appear to be a component of the conserved stress response strategy in land plants [13]. Moreover, the *HRA1* transcript, detected at medium-to-low levels throughout the plant life cycle (Figure S3A), was most strongly induced by short-term oxygen deficiency in plants subjected to a range of abiotic stress conditions (Figure 1A). Hypoxia enhanced the activity of the *HRA1* promoter, as visualized by means of a *promHRA1:GUS* transgenic line (Figure 1B), and led to over 15-fold elevation of *HRA1* mRNA in both the leaves and roots of seedlings (Figure S3B). A survey of our previously generated data of polysome-associated transcripts under the same hypoxia system [15] showed that this was accompanied by active loading of *HRA1* mRNA onto polysomes (Figure S3C), indicating that the synthesis of HRA1 protein occurs during the stress. When *HRA1* expression was monitored over time in hypoxia-treated seedlings, *HRA1* mRNA accumulation was induced rapidly but transiently, whereas that of hypoxia marker *ADH1* increased slowly and steadily during the stress (Figure 1C). This peculiar dynamics of gene expression hinted at a possible role for *HRA1* in the early phase of the low oxygen response.

### HRA1 Represses Gene Transcription During Hypoxia

**Figure 2:**
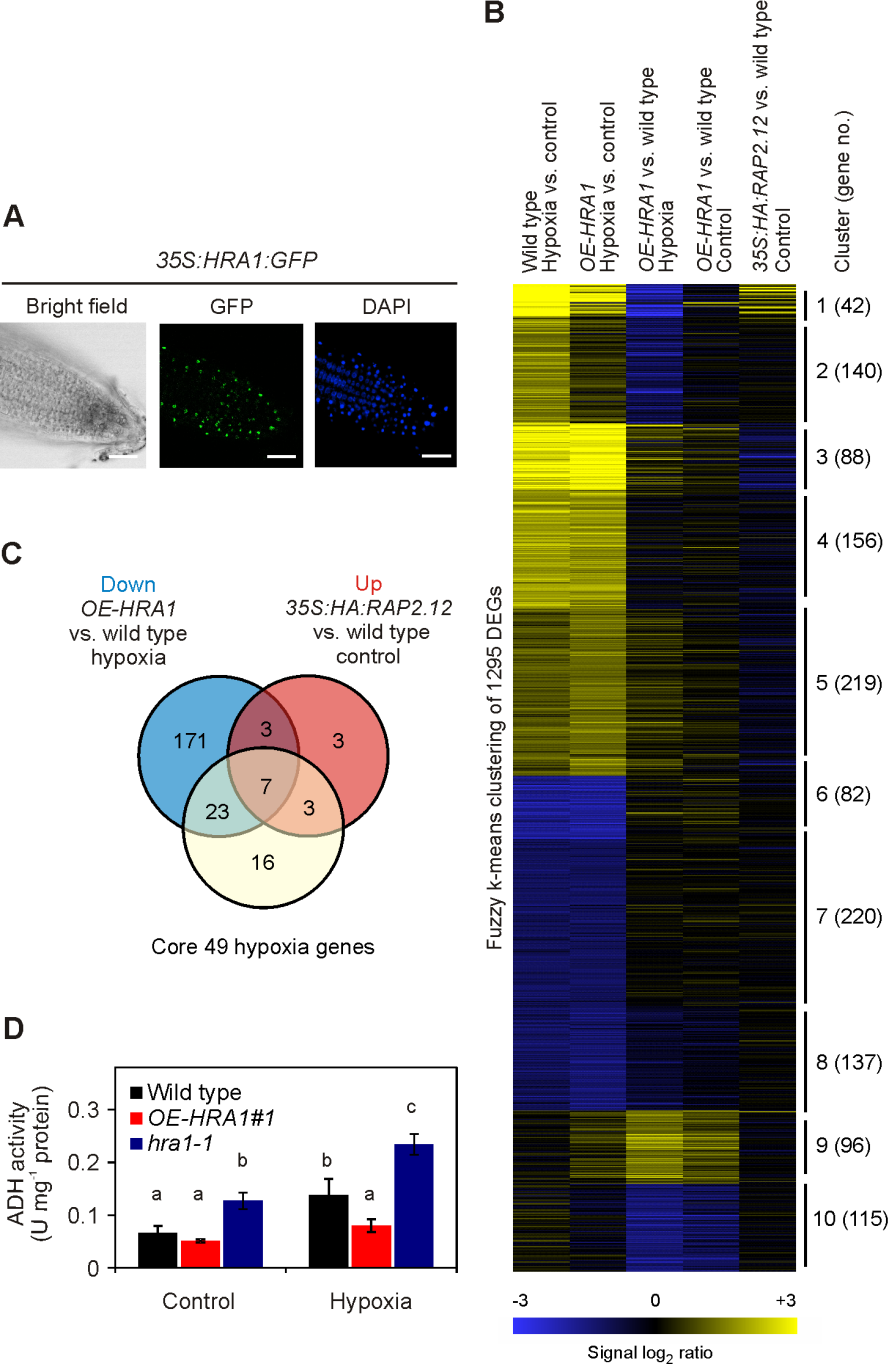
The nuclear factor HRA1 attenuates the expression of hypoxia-responsive genes. (A) Subcellular localization of the HRA1:GFP protein in root cells. Nuclei were visualized by DAPI staining. Scale bar, 30 µm. (B) Differential gene expression in *OE-HRA1* and wild type seedlings under air or hypoxia (2 h), compared with *35S:HA:RAP2.12* transgenics. (C) Venn diagram describing the overlap between genes with opposing regulation by HRA1 or RAP2.12 and 49 genes induced across cell types by hypoxia in wild type seedlings [13]. (D) ADH enzyme activity is affected by altered levels of HRA1 in plants at the seedling stage (mean ± s.d., one-way ANOVA, *p*<0.05, *n* = 3). Hypoxia, 3 h.

A green fluorescent protein (GFP) reporter fusion demonstrated that the HRA1 protein localized to the cell nucleus (Figure 2A), consistent with its prediction as a TF. To investigate the role of HRA1 in transcription, we performed a microarray analysis and compared the hypoxic reconfiguration of the transcriptome between Cauliflower Mosaic Virus *35S:HRA1:FLAG* transgenics (*OE-HRA1*) and wild type Arabidopsis seedlings (Figure 2B and Table S2). Overexpression of *HRA1* significantly reduced the up-regulation of 30 out of the 49 (61%) core hypoxia-responsive genes [13] induced in the wild type after short-term (2 h) hypoxia (|SLR|≥1, FDR<0.01) (Figure 2C), revealing the ability of HRA1 to broadly affect the hypoxic transcriptome. This included genes encoding key enzymes for anaerobic metabolism, such as the marker genes *ADH1* and *PDC1*. The inhibition of hypoxic transcript accumulation by *HRA1* overexpression contrasted to the constitutive up-regulation of the hypoxia-responsive genes observed in *35S:HA:RAP2.12* transgenic seedlings, which constitutively accumulate RAP2.12 due to masking of its N-terminus from the N-end rule machinery [7]. The hypoxic induction of 43% (7/16) of the RAP2.12 up-regulated genes was dampened by ectopic *HRA1* expression (Figure 2C), suggesting there is antagonism between HRA1 and RAP2.12. Consistent with the dramatic reduction in *ADH1* mRNA up-regulation, we found out that ADH activity was significantly repressed in hypoxic *OE-HRA1* seedlings (Figure 2D). It was this squelching of low oxygen induction of many hypoxia-responsive genes that led us to name *At3g10040 HYPOXIA RESPONSE ATTENUATOR 1*.

### HRA1 Mediates Tissue-Specific Responses During Submergence-Induced Hypoxia

**Figure 3:**
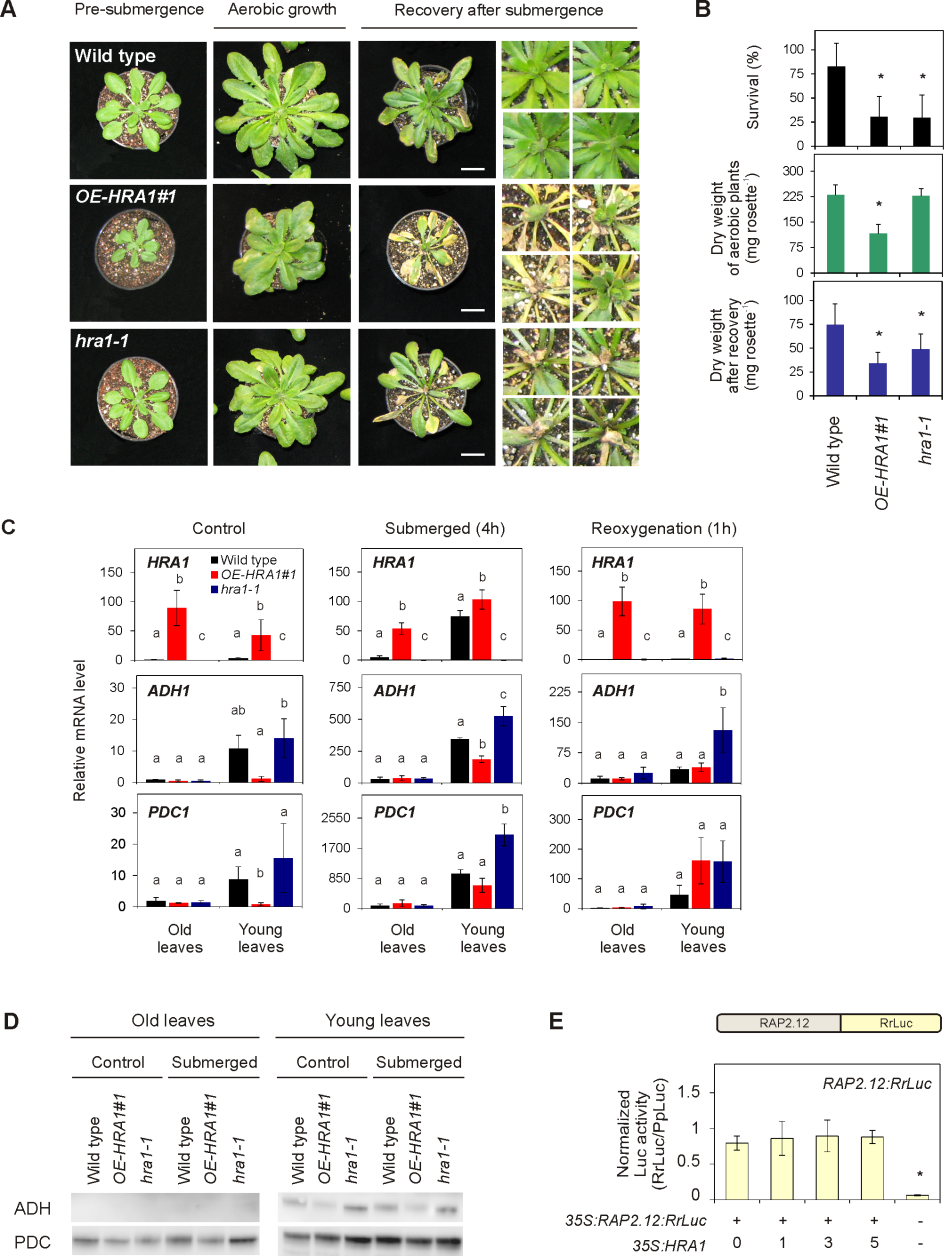
HRA1 contributes to plant submergence survival. (A) Effect of *HRA1* misexpression on rosette growth in air, or after recovery from 72 h submergence in darkness. Scale bar, 2 cm. (B) Percentage of plants surviving flooding-induced hypoxia (*n* = 5), dry weight of rosette plants kept under control growth conditions (*n* = 6), and dry weight of rosettes after postsubmergence recovery (*n* = 6). Data are mean ± s.d.; *p<0.05, significant differences from the wild type after one-way ANOVA. (C) HRA1 regulates target gene transcripts in an age-dependent manner in leaves of plants treated with complete submergence. Transcripts were measured before submergence (“control conditions”), after 4 h submergence in darkness (“submergence”), and after 1 h de-submergence in the light (“reoxygenation”). Relative transcript values were calculated using old leaves of the wild type under control conditions as the reference sample. Data are mean ± s.d. (*n* = 3); letters indicate statistically significant differences between genotypes after one-way ANOVA (*p*<0.05) performed independently on each leaf type. (D) Western blot analysis of ADH and PDC protein accumulation in leaves at different developmental stages from control and submerged (4 h) plants. The full-size images of the hybridized membranes can be found in Figure S10. (E) Stability of the translational fusion RAP2.12:RrLuc protein (RrLuc, *Renilla reniformis* luciferase) in Arabidopsis mesophyll protoplasts upon transfection with increasing amounts of *35S:HRA1*. RAP2.12:RrLuc abundance was evaluated from the RrLuc relative activity, measured through a dual luciferase assay. Data are mean ± s.d. (*n* = 4), and the asterisks indicate statistically significant differences (*p*<0.05) from protoplasts expressing RAP2.12:RrLuc alone, after one-way ANOVA.

The impact of HRA1 on hypoxia-responsive gene expression prompted us to assess how altered levels of *HRA1* expression affect plant performance under submergence-induced hypoxia. We compared two independent *35S:HRA1:FLAG* transgenic genotypes (*OE-HRA1#1* and *#2*) (Figure S4A) and two independent T-DNA insertion homozygous mutants (*hra1-1* and *hra1-2*) (Figures S4B–C and S5) with the wild type. This revealed that both overexpression and failure to produce a full-length *HRA1* transcript reduced the ability of plants to withstand the stress. When tested for tolerance to complete submergence in the dark with two distinct experimental setups, wild type Arabidopsis plants endured the stress significantly longer than *OE-HRA1* plants at the 10-leaf rosette stage (Figure S6A) and, in older rosettes prior to bolting, recovered better than either *OE-HRA1* or *hra1-1* plants (Figure 3A and B). Underwater petiole elongation, a trait recognized as a part of the escape strategy from flooding in semiaquatic species (i.e., deepwater rice and the wetland species *Rumex palustris*) [8], was unaffected by HRA1 (Figure S6B), consistent with previous reports of a limited overall correlation between the trait and flooding survival of mutants in genes up-regulated by hypoxia and Arabidopsis accessions [16],[17]. Moreover, the analysis of the total soluble carbohydrate content in plants prior to submergence allowed us to rule out that a significant difference in the available reserves accounts for the poorer performance of the noticeably smaller *OE-HRA1#1* plants (Figure S7). This was again in line with previous reports of a lack of correlation between carbohydrate content before submergence and stress survival in Arabidopsis [17]. The observation that altered *HRA1* levels modified performance under submergence, at two stages of rosette development and in distinct growth environments, supports the hypothesis of a distinct role for the factor during the stress.

A closer examination of the phenotype of the plants at the end of the recovery period revealed that susceptibility to submergence-induced hypoxia differed in young and older rosette leaves. As compared to the wild type, young leaves emerging from the shoot apex and the shoot apical meristem region were more sensitive in the *hra1-1* mutant, and generally unable to recover during postsubmergence. Contrastingly, the fully expanded and mature leaves of *OE-HRA1#1* plants were more sensitive to dark submergence than the wild type, but the shoot apical meristem performance was less damaged (Figure 3A, see magnified insert). This suggested that HRA1 is imperative for an effective anaerobic response in the meristematic zone and young leaves.

Following preliminary evaluation of temporal regulation of *HRA1* expression in rosette leaves during submergence, which demonstrated an early peak of gene expression after 2 h of stress (Figure S8), we selected 4 h of submergence as a suitable time to study distinctions in gene transcript and protein accumulation in young and fully expanded leaves of wild type and *HRA1* mutant genotypes. Firstly, we then found out that in young leaves, but not in older ones, the expression of the hypoxia marker genes *ADH1* and *PDC1* were differentially regulated by manipulation of *HRA1*. In control and submergence treated plants, *ADH1* and *PDC1* expression was enhanced in *hra1-1* and dampened in young *OE-HRA1* rosette leaves as compared to the wild type (Figure 3C). In all three genotypes, hypoxic gene expression was promptly reversed to presubmergence levels upon reaeration of the plants (Figure 3C, “Reoxygenation”), in agreement with earlier studies [7],[18]. The enhancement in anaerobic gene expression observed in young leaves of *hra1-1* was also seen in the independent *hra1-2* mutant (Figure S9), reinforcing the hypothesis that mutation of *HRA1* leads to altered regulation of the hypoxic response.

Secondly, Western blot analyses performed to detect the products of *ADH1* and all five *PDCs* [19] indicated that elevated levels of these transcripts in young leaves of *hra1-1* plants was accompanied by higher hypoxic production of the encoded proteins (Figures 3D and S10). Although in older leaves some enhancement of PDC accumulation in submergence was visible in the mutant as compared to the wild type, ADH was always below the limit of detection. In younger leaves, contrastingly, ADH protein levels were enhanced in *hra1-1* already under normoxic conditions. These results suggest that HRA1 plays a key role in negatively regulating the induction of *ADH1* and *PDC1* in younger tissues of rosette-stage plants.

We also considered that *HRA1* expression could impact steady-state levels of its upstream regulator RAP2.12. To do so, we evaluated the effect of HRA1 on RAP2.12 protein stability, using mesophyll protoplasts that were transiently transfected with a *35S:RAP2.12:RrLuc* plasmid construct. The stability of RAP2.12 was inferred from the activity of a C-terminal translational fusion of RAP2.12 to the *Renilla reniformis* luciferase (RrLuc) reporter. As RrLuc activity was unaffected by concurrent transfection of the *35S:HRA1* effector, we conclude that HRA1 expression does not affect RAP2.12 stability, at least in isolated leaf protoplasts (Figure 3E). Altogether, these data, presented in Figure 3, support the conclusion that HRA1 acts to limit accumulation of transcripts and the encoded proteins associated with anaerobic metabolism, even in air, particularly in younger rosette tissue.

### HRA1 Contributes to Effective Anaerobic Responses and Normal Plant Development

The submergence survival studies suggested that *HRA1* expression provides vital control of the anaerobic response in the meristematic region and young leaves. To further investigate the spatial and temporal regulation of *HRA1*, we monitored transgenics expressing *promHRA1:GUS*. The beta-glucuronidase (GUS) reporter confirmed that basal *HRA1* promoter activity, detectable under normal growth conditions, was restricted to the shoot apical region and leaf vasculature in aboveground tissues (Figure S11, “Control”), and was pronounced in roots as well (Figure 1B). This pattern of expression is consistent with the hypothesis that HRA1 is active in cells experiencing physiological hypoxia, due to higher oxygen demand, lower permeability to oxygen, or a hypoxic environment [20],[21]. The elevated levels of hypoxia marker gene transcripts in the shoot apical area under normoxia suggest that this region is physiologically hypoxic (Figure 3C), as reported previously [22]. By use of the *promHRA1:GUS* transgenics, we also determined that submergence primarily enhanced GUS activity in the younger rosette tissues and to a much lesser extent in adult leaves, except within the vasculature (Figure S11, “Submergence”).

This pattern of GUS staining correlated well with the tissue-specific effect exerted by *HRA1* on submergence tolerance, as described above (Figure 3A). In genotypes with altered *HRA1* expression, the absence of a fully functional HRA1 protein in *hra1* shoot meristem tissue led to its higher susceptibility to submergence. On the other hand, ectopic expression of *HRA*1 in older rosette leaves of *OE-HRA1* plants reduced their survival of submergence and prolonged darkness, possibly due to accelerated senescence of older leaves (Figure S12).

Our data showed that HRA1 balances low oxygen acclimation responses, but also suggested that proper spatial expression of *HRA1* is required for normal vegetative development. Overexpression of either the native HRA1 protein in *OE-HRA1#3* plants or a FLAG-tagged protein in *OE-HRA1#1* and *#2* plants caused a pleiotropic phenotype that included reduced rosette size, due to shortened petiole length and altered leaf index, slower rosette growth (Figure S13A and B), increased leaf anthocyanin content (Figure S13B), partial loss of apical dominance, delayed flowering, and reduced seed production (Figure S13C). The conservation of these phenotypes across three independent transgenic lines allowed us to recognize their cause in the ectopic expression of high *HRA1* levels in the whole plant, rather than ascribe it to random integration of the transgenes in unrelated genomic loci. We speculate that, although sustained *HRA1* expression is beneficial in rapidly dividing and expanding leaf primordia under normal growth conditions, abnormal HRA1 accumulation in mature leaves has a negative impact on plant development. Moreover, because *hra1-1* and *hra1-2* showed no differences from the wild type under normal growth conditions (Figure S13), we can conclude that mutations in the 3′ region of the *HRA1* transcript (Figure S4B and S5) particularly affect the hypoxic pathway (Figure 3A–D) but do not relate to the developmental phenotypes shown here.

### HRA1 Associates with Few Differentially Regulated Genes in Hypoxic Seedlings

**Figure 4:**
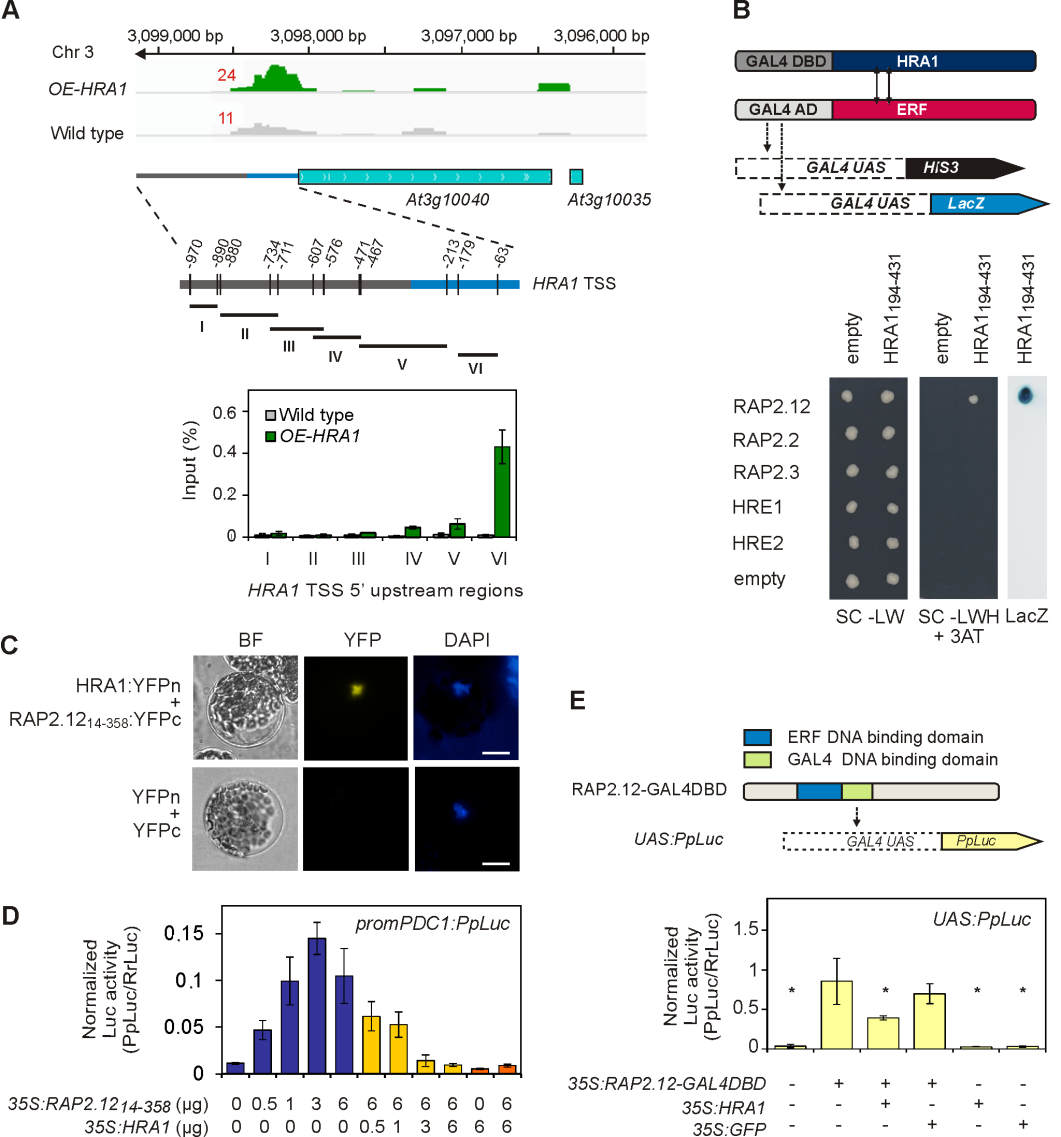
HRA1 modulates transcription of anaerobic genes through protein–protein interaction with RAP2.12. (A) HRA1 binding site on the upstream region of the *HRA1* gene. The number next to the peak indicates the peak summit. ChIP-seq peak area for *HRA1* was subsequently divided into six regions for confirmation of DNA binding using ChIP-qPCR (see Table S4 for primer sequences). TSS, transcription start site. (B) Yeast-two-hybrid assay between an HRA1 C-terminal fragment (HRA1_194–431_) and the five Arabidopsis ERF-VII proteins. AD, activation domain; DBD, DNA binding domain; UAS, upstream activating sequence. SC-LW, control medium −Leu −Trp; SC-LWH+3AT, selective medium −Leu −Trp −His +3AT; LacZ, β-galactosidase assay. (C) Bimolecular fluorescence complementation between HRA1 and RAP2.12 in Arabidopsis mesophyll protoplasts. DAPI staining indicates the position of the nucleus. Scale bar, 20 µm. (D) Transcriptional activation of the *PDC1* promoter, visualized through a firefly *luciferase* (*PpLuc*) reporter fusion, by RAP2.12 alone (blue bars) and in combination with HRA1 (yellow bars) or its C-terminal fragment HRA1_194–431_ (orange bars). Data are mean ± s.d. (*n* = 4). (E) Dual luciferase assay showing that HRA1 repression of RAP2.12 is independent of HRA1 binding to DNA. A heterologous promoter made up of four repeats of the yeast *GAL4* upstream activating sequence (“*GAL4 UAS*”) was introduced into plant protoplasts and could only be recognized by chimeric GAL4DBD (GAL4 DNA binding domain)-containing TFs, in this case by RAP2.12-GAL4DBD [7]. GFP was used as a negative control, as a RAP2.12 noninteracting protein. Data are mean ± s.d. (*n* = 3); *p<0.05, statistically significant difference from the positive interaction produced by RAP2.12-GAL4DBD.

To gain insight into the role of HRA1 dampening core hypoxia gene transcription during hypoxia, we performed chromatin immunoprecipitation followed by deep sequencing (ChIP-Seq analysis) using seedlings deprived of oxygen for 2 h, in the same manner as the transcriptome analysis. To identify chromatin bound by HRA1-FLAG, *OE-HRA1#1* and Col-0 seedling tissue was cross-linked, nuclei were isolated, and immunopurification performed with a FLAG antibody. The Col-0 sample was used as a control to monitor nonspecific immunopurification. Deep sequencing of ∼100 bp fragments yielded 146 peak-to-gene associations (Table S3), corresponding to putative HRA1 target genes, 42% of which (62 elements) fell 5′ of the predicted transcription start sites (Figure S14A). We then focused on the 1,295 differentially expressed genes (DEGs) in the microarray dataset and found out that seven of the HRA1 binding sites resided on genes significantly regulated by hypoxia and/or HRA1 overexpression (*HRA1*; *RAV1*, *At1g13260*; *HUP7*, *At1g43800*; a hydroxyproline-rich glycoprotein family protein coding gene, *At1g31310*; *WRKY7*, *At4g24240*; *CYP78A6*, *At2g46660; DCL1*, *At1g01040*) (Figure S14B). The small number of stress-responsive genes identified by ChIP of HRA1 suggests that its effect on the hypoxia-responsive gene transcription may be mediated by other DNA binding factors, rather than HRA1's ability to recognize DNA. Finally, of the candidate targets of HRA1, only *HUP7* and *HRA1* itself (Figure 4A and Table S4) were constitutively up-regulated by HA-RAP2.12 and less hypoxia-induced in *OE-HRA1* plants (Figure S14C). This led us to consider that repression of hypoxia-responsive genes by HRA1 was largely independent of its direct association to chromatin of the genes it regulates.

### HRA1 Directly Interacts with the ERF-VII TF RAP2.12

We hypothesized that HRA1 could attenuate hypoxia-responsive gene expression by directly inhibiting RAP2.12 activity. To address this, we first examined whether protein–protein interaction occurs between HRA1 and RAP2.12. Previously, interaction between rice GTγ-clade trihelix protein LOC_Os11g06410 (SAB18) (Figure S2) and the ERF-VII TFs SUBMERGENCE1A (SUB1A) and the related SUB1C (LOC_Os09g11460) was reported in a nondirected yeast-two-hybrid screen [23]. We confirmed that HRA1 and RAP2.12 interact in the heterologous yeast-two-hybrid system (Figures 4B and S15A–C). By systematically testing different combinations of full-length and truncated versions of HRA1 and RAP2.12 (Figure S15A), we determined that, firstly, the conserved C-terminal region rather than the trihelix domain of HRA1 was required for RAP2.12 association (Figures 4B and S15B) and, secondly, the N-terminal portion of the ERF-VII (RAP2.12_1–123_) was sufficient for interaction (Figure S15C), while its DNA binding domain might enhance the association (Figure S15B). The interaction between HRA1 and RAP2.12 was subsequently validated *in planta* by means of bimolecular fluorescence complementation using Arabidopsis protoplasts (Figure 4C).

We also tested HRA1 interaction with the other four Arabidopsis ERF-VII factors in the yeast-two-hybrid system (Figure 4B). Interestingly, only RAP2.12 interacted with the HRA1 protein, consistent with the fact that the RAP2.12 1–123 region, which was sufficient for binding, is poorly conserved among Arabidopsis ERF-VII sequences (with the exception of the N-terminal region, critical to N-end rule regulation, which was anyway not required for HRA1 interaction *in planta*; Figure 4C). It remains an open question why no interaction was observed with RAP2.2, which has the highest level of sequence similarity with the RAP2.12 protein in the interaction region.

### HRA1 Modulates the Activity of the ERF-VII TF RAP2.12

With the knowledge that HRA1 and RAP2.12 interact, we investigated if the interaction could account for HRA1-mediated attenuation of RAP2.12-driven transcriptional activation. Towards this goal, we used a firefly *luciferase* (*PpLuc*) reporter fusion to measure the activity of the RAP2.12-responsive *PDC1* promoter (−911 to −1 relative to the start codon) in transiently transfected Arabidopsis mesophyll protoplasts. We confirmed that *35S:RAP2.12_14–358_* effector plasmid DNA enhanced the luciferase activity of *promPDC1:PpLuc* (Figure 4D). When increasing amounts of *35S:HRA1* were co-transfected, the luciferase activity gradually fell towards basal levels, indicating that RAP2.12 potential in *promPDC1:PpLuc* transactivation was negatively affected by HRA1, presumably due to the interaction between factors. Inhibition of *promPDC1:PpLuc* expression was similarly achieved by concurrent expression of *35S:HRA1_194–431_* (Figure 4D), which lacked the trihelix DNA binding domain (Figure S15A) but retained the C-terminal region that allowed interaction with RAP2.12 in the yeast-two-hybrid assay (Figures 4B and S15B). This demonstrates that the repression of RAP2.12 activation of *PDC1* transcription by HRA1 was independent of the trihelix domain and, therefore, most likely the TF's binding of DNA. This is consistent with the absence of *PDC1* and many other HRA1-regulated genes in the immunoprecipitated chromatin.

To further validate the hypothesis that HRA1 inhibits RAP2.12 through direct interaction rather than DNA binding, we took advantage of an artificial *UAS* promoter, made up of four repetitions of the yeast *GAL4* upstream activating sequence [7], which cannot be recognized by endogenous plant factors. By this approach we confirmed that the activation of the *UAS:PpLuc* construct by a chimeric RAP2.12-GAL4DBD factor was inhibited by coexpression of *35S:HRA1* in protoplasts, in spite of the inability by HRA1 to recognize the *UAS* promoter (Figure 4E). Altogether, these observations demonstrated that HRA1 inhibits RAP2.12 function by direct protein–protein interaction, rather than by competition for DNA binding.

Additional evidence of the impact of RAP2.12 inhibition by HRA1 was obtained in protoplasts, whose survival of hypoxia was enhanced by transfection with *35S:RAP2.12* only if *35S:HRA1* was not concurrently transfected (Figure S16A), and *in planta*, where overexpression of a stabilized RAP2.12_14–358_ protein in the *OE-HRA1#1* background was sufficient to suppress the alteration in *OE-HRA1* rosette morphology and return the overall phenotype to that of the wild type (Figure S16B).

### HRA1 Is Regulated by a Negative Feedback Mechanism

**Figure 5:**
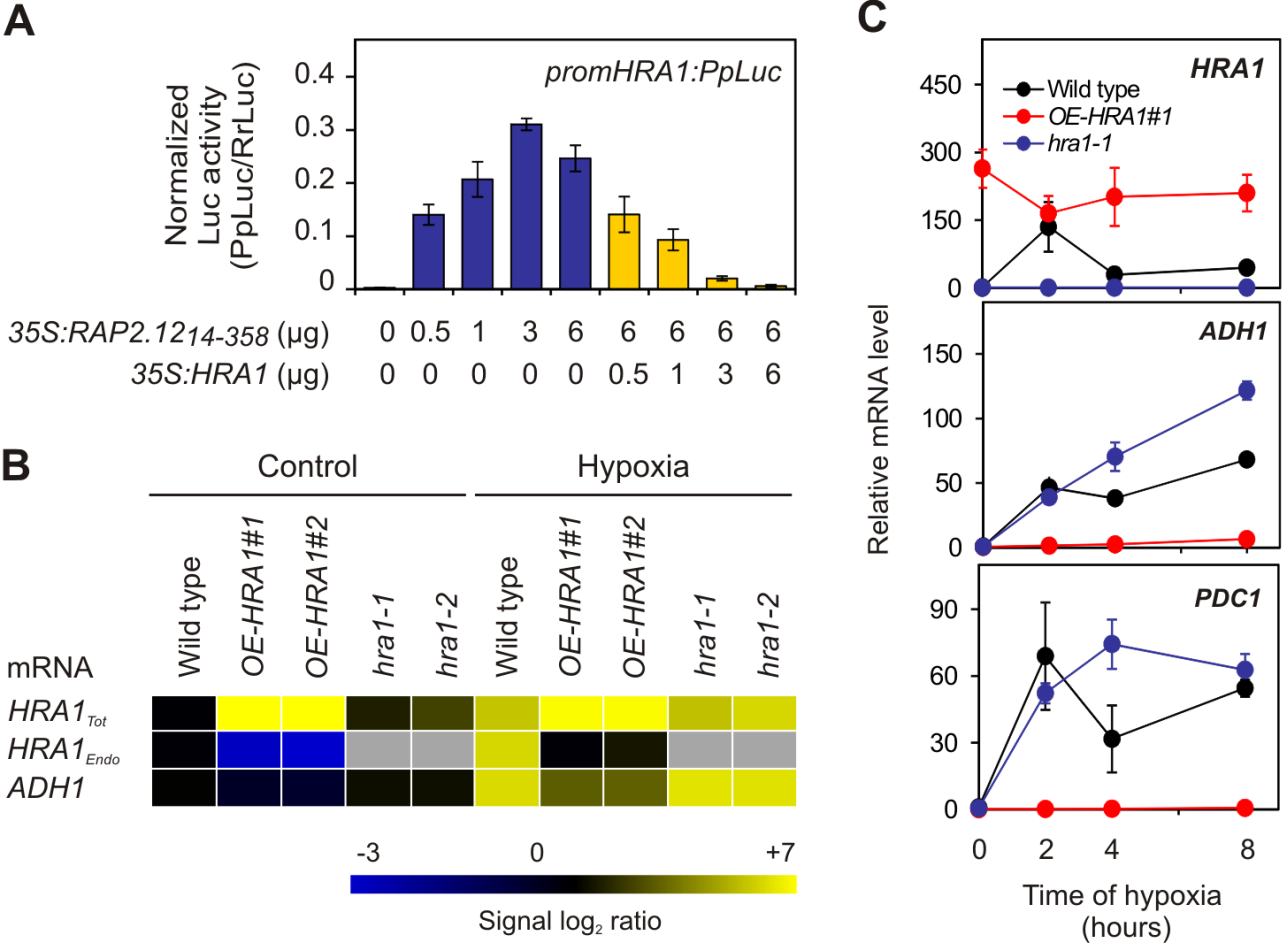
A negative feedback loop acting on HRA1 determines the extent of anaerobic gene expression over the time of stress. (A) Modulation of *HRA1* promoter activity, as visualized through the firefly luciferase reporter, by co-transfection of protoplasts with a stabilized RAP2.12_14–358_, alone (blue bars) or in combination with a HRA1 effector construct (yellow bars). Data are mean ± s.d. (*n* = 4). (B) Steady-state levels, in Arabidopsis seedlings, of the full-length *HRA1* mRNA (*HRA1_Endo_*), measured with specific *HRA1 3′-UTR* primers, and of full-length (in the wild type), truncated (in *hra1-1* and *-2*), and overexpressed transcripts (in *OE-HRA1-#1* and *-#2*) (*HRA1_Tot_*), measured with primers specific for *HRA1* coding sequence; see Figure S4B for the position of primers used for *HRA1_Tot_* and *HRA1_Endo_* mRNA abundance measurement. Hypoxia, 2 h. Data are mean ± s.d. (*n* = 3 ). The absence of expression is indicated by grey rectangles (masked). Numeric expression values are provided in Table S5. (C) Abundance of *HRA1* and hypoxia marker gene mRNAs over prolonged hypoxia stress in *OE-HRA1* and *hra1* seedlings. Data are mean ± s.d. (*n* = 4).

Because the molecular response to hypoxia might entail a balance between RAP2.12 stabilization and attenuation under low oxygen stress, tight regulation of HRA1 was anticipated. As for the promoter of *PDC1*, we found that the *HRA1* promoter (−849 to −1 relative to the start codon) was transactivated in a dosage-dependent manner by RAP2.12 and repressed by HRA1 itself in mesophyll protoplasts (Figure 5A). An additional mechanism contributing to *HRA1* regulation involves binding of HRA1 to its own promoter, as revealed by ChIP-Seq and confirmed by ChIP-qPCR (Figure 4A). We hypothesize that this binding dampens RAP2.12 activation of this promoter. In support of this, when specific RT-qPCR was performed to detect the expression of the endogenous *HRA1* gene (Figure S4B), strong down-regulation was observed in *OE-HRA1* plants (Figure 5B). These results demonstrate that *HRA1* transcription is activated upon hypoxia following the nuclear accumulation of RAP2.12 but is subsequently subjected to negative self-regulation. This can be considered a “double check” mechanism that takes advantage of HRA1's ability to both repress RAP2.12 activity and directly bind its own promoter, possibly competing with RAP2.12 binding. The double regulation of *HRA1* transcription is most likely responsible for the transient dynamics of *HRA1* transcript accumulation during hypoxia (Figure 1C) and allows the plant to limit hypoxic gene expression over time, as detected in the wild type and to a lesser extent in the *hra1-1* mutant (Figure 5C).

## Discussion

Gene expression is tightly regulated in response to low oxygen stress. In order to maximize the efficiency of ATP utilization, the transcription of many genes, whose function is not essential for survival, is repressed under low oxygen stress, whereas polyribosomes dissociate from their mRNA to limit translation [18],[24]. At the same time, the metabolism of plants is adapted to hypoxia through a reconfiguration of the energetic pathways that enables fermentation to maintain substrate-level ATP production through glycolysis after replacement of the oxidative phosphorylation [1],[8]. This requires transcriptional activation of genes such as *PDC1* and *ADH1*, encoding essential enzymes for ethanolic fermentation. Although the transcriptional rearrangement following exposure to hypoxia is not limited to the expression of fermentation-related genes, this pathway contributes largely to survival in low oxygen conditions, as mutants lacking *PDC* and *ADH* genes are hypersensitive to hypoxia and conditions with a hypoxic component [4]–[6]. Moreover, the transcriptional induction of *ADH* and *PDC* genes is a conserved feature in the anaerobic response of all higher plants studied so far [13].

Transcriptional activation of fermentative genes is downstream of the oxygen-sensing machinery, which relies on the N-end-rule–dependent stabilization of the ERF-VII TFs, such as RAP2.12 [1]. However, this mechanism may not be an on–off process but rather modulated in intensity by additional hypoxic players, as both environmental fluctuations in oxygen availability [25], as well as local hypoxic microenvironments in developing tissues and organs [20],[26], may necessitate temporal and spatial flexibility in the hypoxic response. This is because stabilization of RAP2.12 would trigger activation of the core hypoxia genes, with their down-regulation reliant upon reoxygenation and the destabilization of RAP2.12. Such inflexibility could expose cells to unregulated fermentative metabolism that may rapidly exhaust the limited respiratory substrates [27],[28], preventing endurance of prolonged stress and limiting recovery upon reoxygenation. Here, we show that the strategy adopted by cells to respond to decreased oxygenation entails the induction of a repressor of hypoxic gene expression, the nuclear-localized trihelix protein HRA1, and confirm this protein acts as a direct attenuator of the low oxygen stabilized transcriptional activator RAP2.12 in Arabidopsis.

It cannot be excluded that HRA1 may mediate additional mechanisms of repression, starting from the cascade activation of hypoxia-specific transcriptional repressor(s), either at the transcriptional or post-translational level of regulation (*i.e.*, activation of a repressor *via* protein-protein interaction). The absence of candidate transcriptional repressors among HRA1 targets (according to our microarray and ChIP-seq analyses), along with HRA1's ability to restrain anaerobic promoter activation even after ablation of its DNA binding domain (Figure 4D), supports the conclusion that attenuation of RAP2.12 by HRA1 is not accomplished through DNA binding. Contrastingly, HRA1's ability to bind its own promoter appears to provide a second tier of activity, namely inhibition of its transcription.

The present study expands the knowledge of the hypoxia-response transcription network mediated by the low oxygen stabilized ERF-VIIs. The fast induction of *HRA1*, notably directed by RAP2.12 at the onset of hypoxia, confers the ability to prevent excessive expression of anaerobic genes, particularly in younger tissues exposed to submergence (Figure 3C). The up-regulation of the attenuator HRA1 serves to limit the activity of stabilized RAP2.12. This may enable the cells expressing HRA1 to limit carbon catabolism through fermentation, conserving energy reserves required at the restoration of normoxia. Interestingly, in *SUB1A*-containing varieties of rice the ability to resume meristem development upon desubmergence is linked to an energy-saving quiescence strategy associated with submergence tolerance [28]. The recognition of a trihelix protein that interacts with SUB1A in rice [23] leads to the question whether the regulation of plant ERF-VIIs may broadly rely on trihelix-dependent attenuation mechanisms similar to the one we described in Arabidopsis.

**Figure 6:**
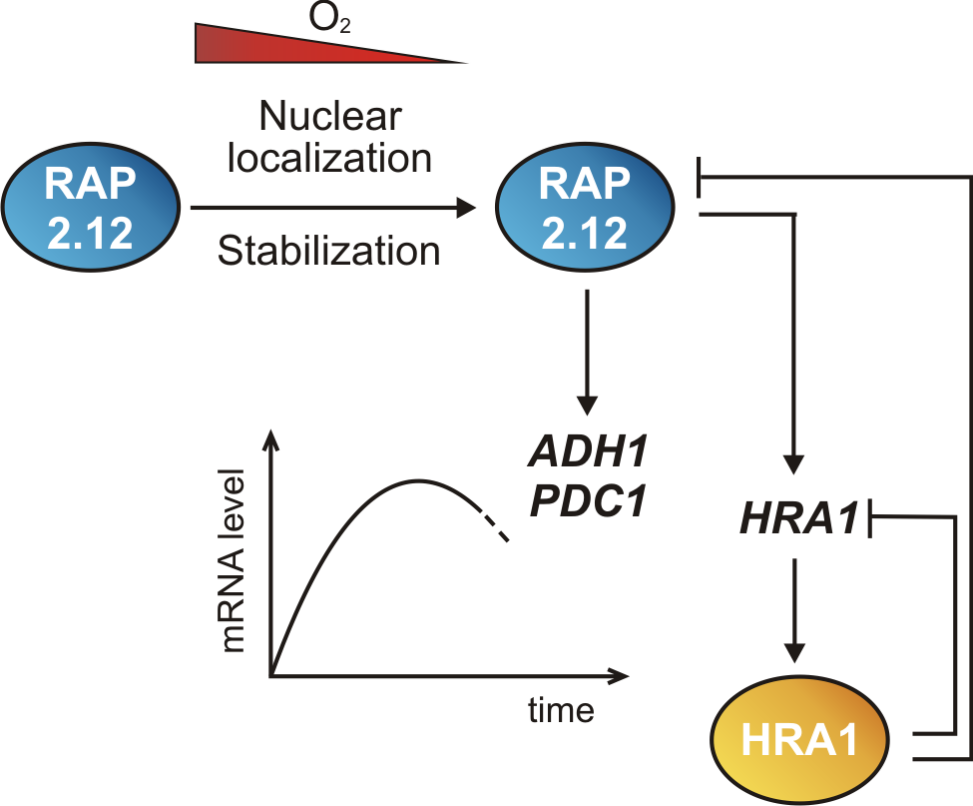
Model summarizing how the balanced action of RAP2.12 and HRA1 tunes transcription of hypoxia target genes. In plant cells, hypoxia promotes the relocalization of RAP2.12 to the nucleus, which triggers the expression of *HRA1* and other RAP2.12 target genes. Once synthesized, the encoded HRA1 protein generates two negative loops of feedback regulation, one acting on RAP2.12 and another on HRA1 itself. The first one dampens RAP2.12 activity, thereby limiting the anaerobic gene expression after its initial burst. The negative self-regulation is, instead, supposed to contribute to the subsequent down-regulation of HRA1 and makes a later wave of anaerobic gene expression possible under prolonged hypoxia.

We show that HRA1 acts through a sophisticated mechanism that involves physical interaction with RAP2.12 to down-regulate its transactivation capacity and generates a feedback loop of negative self-regulation (Figure 6). This latter mechanism may make it possible for the cell to start a new pulse of gene expression if hypoxia is prolonged. It is important to highlight that HRA1 interacts with RAP2.12, but apparently not with HRE1 and HRE2. This is suggestive of a hierarchy in the involvement of ERF-VIIs in the anaerobic response, with the initial burst resulting from the action of RAP2.12 and HREs taking over during prolonged hypoxia, in line with the hypoxia susceptibility of *hre1hre2* double mutants corresponding to their inability to sustain the expression of the hypoxia-responsive genes [29]. The relative contribution of the different ERF-VIIs requires further exploration and will likely reveal additional layers of complexity of the anaerobic transcriptional response network.

The tissue-specific expression of HRA1 unveils the importance of differential modulation of the anaerobic response in rosette leaves of distinct developmental age. *HRA1* is predominantly expressed in tissues prone to physiological hypoxia and mutants lacking HRA1 display organ-specific susceptibility to hypoxia (Figure 3A). This implies that the need for a finely-tuned hypoxic response is varied across organs and during development. Fast growing tissues, such as young expanding leaves, required HRA1-dependent dampening of the anaerobic response for survival, and this is likely related to the need to preserve resources for resumption of growth following reoxygenation. Mature leaves, instead, can devote available carbon to fuel fermentation to preserve leaf tissue homeostasis, with less requirement for biosynthetic processes. Carbon will be more rapidly available at reoxygenation by resuming of the photosynthetic activity in source leaves, as compared to younger, sink leaves.

Although plants possess a vascular system for transporting nutrients, its ability to transport oxygen relies on anatomical features, such as aerenchyma, that are absent in many instances. Physiological and molecular acclimation to rapidly changing oxygen availability, due to environmental perturbations such as flooding or on a daily basis as a consequence of the light/dark cycle, requires a sophisticated mechanism to fine-tune the anaerobic response. We can conclude that two components of this system in Arabidopsis are the N-end-rule–regulated ERF-VIIs and the trihelix HRA1. It is well established that genetic variation of ERF-VIIs in rice confer distinct survival strategies and manipulation of these proteins in Arabidopsis can be used to bolster low oxygen and submergence survival. Our evidence of a mechanism regulating the efficacy of the RAP2.12-dependent transcriptional regulation provides experimental support for the existence of an elaborated system allowing the plants to respond dynamically to hypoxia. This mechanism is based on the equilibrium between the induction of the anaerobic response by group VII ERFs and repression by HRA1. Alteration of this equilibrium by misexpression of HRA1 results in lower tolerance to submergence, suggesting that crops with higher tolerance to flooding conditions might be bred through fine-tuning of the relative contribution of ERFs and HRA1 to the overall response to hypoxia. The presence of HRA1 orthologues in crops provides additional opportunity for engineering or breeding varieties with enhanced tolerance to flooding.

## Materials and Methods

### Plant Material

*A. thaliana* Columbia-0 (Col-0) was used as the wild type ecotype. *hra1-1* (N541486; SALK_041486) and *hra1-2* (N560275; SALK_060275) T-DNA mutants were obtained from the European Arabidopsis Stock Center (uNASC) and the Arabidopsis Biological Resource Center, respectively. Mutants were genotyped using standard nonquantitative PCR on genomic DNA, using primers listed in Table S7. See also Figure S4B for a graphical representation of primer binding sites.

### Growth Conditions

Seeds were sown in a moist mixture of soil∶perlite∶sand mixture 3∶1∶1, stratified at 4°C in the dark for 48 h and germinated at 23°C day/18°C night under a neutral day cycle (12 h light/12 h darkness, ∼80 µmol photons m^−2^ s^−1^ light intensity). Experiments in sterile conditions were performed with 4-d-old seedlings grown in liquid MS medium [0.43% (w/v) Murashige–Skoog (MS) salts (Sigma-Aldrich), 1% (w/v) sucrose, pH 5.7] under continuous shaking conditions, or with 2- and 3-wk-old plants grown vertically on solid MS medium [liquid MS medium, 0.4% (w/v) Phytagel (Sigma–Aldrich)]. For the DNA microarray, chromatin immunopurification, and ADH assay experiments, sterilized seeds were grown for 7 d on solid MS medium in vertical orientation in a growth chamber (Model # CU36L5, Percival Scientific, Perry, IA) under a long day cycle (16 h light/8 h darkness, ∼80 µmol photons m^−2^ s^−1^), at 23°C, before treatments [18].

### Low Oxygen Treatments

Hypoxic treatments were performed as described previously [18].

For submergence treatments, 4-wk-old plants (stage 3.50 [30]) grown in soil as described above were used. Treatments started at ZT (Zeitgeber Time) 2. Plants were submerged with deionized water in glass tanks, until the water surface reached 20 cm above the rosettes, and kept in the dark for the duration of the treatment. Submergence was for 72 h, after which plants were transferred to normal photoperiodic conditions (12 h light/12 h darkness) and allowed to recover for 1 wk before the phenotypic evaluation. Plants that were able to progress in vegetative development were scored as survivors (Figure 3A). The dry weight of whole rosettes was measured before submergence and at the end of the recovery phase. Five separate tanks were used in every submergence experiment, each containing five plants per genotype, and the experiment was repeated three times.

Samples for gene transcript abundance and Western blot analyses were, instead, collected after 4 h of submergence. Each sample was composed of young leaves (youngest three emerging leaves and shoot meristem) or old leaves (10th to 12th leaves) from five plants. Three biological replicates were used, and the experiment was repeated two times with comparable results. Gene expression data are mean ± s.d.

In an independent submergence survival assessment system [16],[17], seedlings at the 10-leaf rosette stage (stage 1.10 [30]) were submerged in complete darkness or held in complete darkness in air for 3, 5, 7, or 10 d (Figure S6). After desubmergence or re-illumination, the number of plants with alive apical meristem (green, nonwater-soaked) was recorded each day for 12 d. The median lethal time (LT_50_), standard error, and the 95% confidence interval were determined using the 9-d recovery time point exactly as described previously [17].

### Other Stress Treatments

Additional abiotic stress treatments were carried out on liquid-grown 4-d-old seedlings. The following conditions were used: 2 h at 4°C (cold stress); pinching of the seedlings with 10 consecutive pin pricks, 1 h before sampling (mechanical wounding); 3 h in the presence of 150 mM sodium chloride (salt stress); 3 h in the presence of 5 mM hydrogen peroxide (oxidative stress); 3 h in the presence of 100 mM mannitol (osmotic stress); 3-h-long desiccation under laminar air flux (dehydration stress); 90 min at 38°C (heat stress). Control plants were maintained at 23°C with continuous shaking.

### Cloning of Constructs

Coding and upstream regulatory sequences were amplified from appropriate Arabidopsis cDNA or genomic DNA templates using Phusion High Fidelity DNA-polymerase (New England Biolabs). Fusion sequences were generated by overlapping PCR. Whenever the GATEWAY cloning system (Life Technologies) was exploited, sequences were cloned into pENTR/D-TOPO and the resulting entry vectors were recombined into destination vectors using the LR reaction mix II (Life Technologies). A list of plasmid constructs generated in this study and primers used for cloning can be found in Tables S6 and S7, respectively.

A construct for overexpression of *HRA1* in the *OE-HRA1#1* and *OE-HRA1#2* transgenics, named *35S-HRA1-FLAG*, was prepared by cloning the full-length *HRA1* cDNA with gwHRA1_5′UTR_Fw and gwHRA1_3′UTR_Rv primers and recombination into the p35S:GATA-HF vector [15], in which a CaMV 35S promoter drives the expression of *HRA1* cDNA linked to a C-terminal FLAG tag [NH2-Gly7-FLAG(AspTyrLysAsp4Lys)Gly3-His6-COOH]. A further *35S-HRA1* overexpression construct, used to obtain a third transgenic lacking any C-terminal epitope tag (*OE-HRA1#*3), was produced by amplification of *HRA1* coding sequence with gwHRA1_Fw and gwHRA1_Rv primers and subsequent cloning in the pK7WG2 vector [31].

The *35S:RAP2.12:RrLuc* construct exploited for Figure 3E was produced by GATEWAY cloning of a *RAP2.12:RrLuc* DNA sequence into p2GW7; this sequence, in turn, was produced by overlapping PCR after separate amplification of the *RAP2.12* full CDS, from a cDNA template, and *Renilla reniformis luciferase* CDS, from the *35S:RrLuc* plasmid (see Table S6). Moreover, the normalization vector *35S:PpLuc* was generated by amplification of the firefly *luciferase* gene from pBGWL7 [31] and GATEWAY cloning into p2GW7.

Finally, the pGWL7 GATEWAY destination vector used for transactivation experiments in plant protoplasts was obtained by cutting an ApaI/SpeI fragment from pBGWL7 and ligating it into the p2GW7 backbone [31].

### Plant Transformation

Stable transgenic Arabidopsis plants were generated by Agrobacterium-mediated transformation following the floral dip method [32]. T_0_ seeds were screened on the appropriate selection plates, and single-insertion homozygous lines were identified. T_3_ or later generations of single insertion homozygotes were evaluated.

### Bimolecular Fluorescence Complementation and Reporter Transactivation Assays Using Protoplasts

Arabidopsis mesophyll protoplasts were obtained from rosette leaves and transfected according to [33].

*In planta* protein–protein interactions were investigated via bimolecular fluorescence complementation (BiFC) [34], using the C-terminal split-YFP constructs *35S:HRA1:YFPn* and *35S:RAP2.12_14–358_:YFPc* [35]. As the negative control for nonspecific YFP complementation, empty *35S:YFPn* and *35S:YFPc* vectors were co-transfected into protoplasts. For each construct, 10 µg plasmid DNA was used. Fluorescence was observed with a Nikon ViCo microscope using filters for YFP (excitation wavelengths, 495–510 nm; barrier, 520–550 nm), TRITC (excitation wavelengths, 540–565 nm), and DAPI (excitation wavelengths, 385–400 nm). Micrographs are representative of three independent experiments.

In promoter transactivation assays performed with protoplasts, 3 µg transformation^−1^ *35S:RrLuc* plasmid DNA [36] was used for normalization of the PpLuc activity. Test constructs (test *promoter:PpLuc*, 3 µg transformation^−1^) harboring the *Photinus pyralis luciferase* gene were co-transfected into protoplasts, along with the specified effector plasmid(s) encoding TFs (*35S:effector*, up to 6 µg transformation^−1^). pAVA 393 [37] was used as the *35S:GFP* construct, when needed. Samples were subsequently processed with the Dual-Luciferase Reporter Assay System (Promega), and luciferase activity was quantified with a Lumat LB 9507 luminometer (Bechtold Technologies). Each experiment was repeated three times and a representative replicate was shown. Relative luciferase intensity values (PpLuc/RrLuc) are presented as mean ± s.d. of four independent transfections.

RAP2.12:RrLuc protein stability from Figure 3E was assessed likewise through the Dual-Luciferase system. For each individual transfection, 5 µg *35S:RAP2.12:RrLuc* plasmid DNA was supplemented with increasing amounts of the *35S:HRA1* effector construct and transfected into a mesophyll protoplast suspension. In this case, *35S:PpLuc* was used for normalization. Relative Renilla luciferase intensity values (RrLuc/PpLuc) are presented as mean ± s.d. of four independent transfections.

### Localization of GFP in Planta

For HRA1 localization in plant tissue, a *35S:HRA1:GFP-His6-FLAG* translational fusion construct (named *35S-HRA1-GFP*), was generated by subcloning of a *HRA1:GFP-His6* construct, obtained by recombining the full-length *HRA1* cDNA in the pEarleyGate103 vector [38], into the p35S:GATA-HF plasmid [15]. The construct was transformed into Arabidopsis Col-0 to produce transgenic plants accumulating the HRA1:GFP protein. Three-day-old seedlings were vacuum infiltrated with 5 µg ml^−1^ 4′, 6-diamidino-2-phenylindole (DAPI) for 10 min, washed in water for 10 min under vacuum, and observed with a Leica SP2 (Bannockburn, IL) confocal microscope at the Microscopy Core Facility, Institute for Integrative Genome Biology, University of California, Riverside. GFP was viewed by excitation at 488 nm and emission at 500–600 nm. DAPI stained nuclei were visualized with a UV laser by excitation at 350 nm and emission at 399–600 nm.

### GUS Staining

Histochemical GUS staining was carried out according to [39]. Briefly, plant material was fixed immediately after sampling in ice-cold 90% acetone for 1 h, rinsed several times in 100 mM phosphate buffer (pH 7.2), and then stained in a freshly prepared reaction solution [0.2% Triton X-100, 2 mM potassium ferrocyanide, 2 mM potassium ferricyanide, and 2 mM X-Gluc (5-bromo-4-chloro-3-indolyl ß-D-glucuronide, sodium salt dissolved in DMSO) in 100 mM phosphate buffer pH 7.2]. Plants were stained for 2–4 h (seedlings) or overnight (adult plants). Chlorophyll was eliminated from green tissues by washing them with absolute ethanol.

### RT-qPCR

RNA extraction, removal of genomic DNA, cDNA synthesis, and RT-qPCR analyses were performed as described previously [36]. Steady-state mRNA levels were normalized using *Ubiquitin10* (*At4g05320*) or *β-TUB2* (*At5g62690*) as reference genes and relative expression values were calculated using the comparative Ct method [40]. The complete list of qPCR primers employed is reported in Table S8. Multiple qPCR primer couples were designed on *HRA1*-derived transcripts: Those named “sgHRA1_Endo” and “sgHRA1_Tot” were exploited to measure *HRA1* mRNA levels in Figure 5B, while elsewhere “sgHRA1” primers were used. Data (mean ± s.d.) are representative of at least two independent experiments, each one carried out with three biological replicates, unless differently stated.

### Microarray Analysis

Total RNA was extracted from frozen tissue using the RNeasy Plant Kit (Qiagen, Chatsworth, CA) and quantified with a NanoDrop 1000 spectrophotometer (ThermoScientific, Wilmington, DE). RNA quality was checked using the Agilent 2100 Bioanalyzer (Santa Clara, CA), and biotin-labeled cRNA was prepared with the GeneChip IVT Labeling Kit (Affymetrix, Santa Clara, CA). Hybridizations against the Arabidopsis ATH1 Genome Array were performed by the Institute for Integrative Genome Biology (IIGB) Genomics Core Facility, University of California, Riverside. Transcriptomes of each of the three genotypes—namely, Col-0, *OE-HRA1#1*, and *OE-HRA1#2*—were profiled under the two conditions. CEL files of two OE lines were processed as biological replicates along with two Col-0 replicates, using R and Bioconductor package. The NCBI Gene Expression Omnibus (http://www.ncbi.nlm.nih.gov/geo/) accession number for the generated dataset is GSE50679. The microarray dataset generated by [7] was obtained from NCBI GEO (http://www.ncbi.nlm.nih.gov/geo/) accession no. GSE29187. CEL files of aerobic-treated 5-wk-old rosette tissues of Col-0 and a transgenic overexpressing N-terminally HA-tagged RAP2.12 (RAP) line were processed together with the HRA1 microarray dataset (Col-0, *OE-HRA1#1*, and *OE-HRA1#2*). After computing the absent and present calls using the Affymetrix MAS 5.0 algorithm [41], datasets were normalized using the robust multichip average (RMA) method [42]. Mitochondrial and plastid gene probe pair sets were removed, and probe pair sets with present calls in greater than 50% of the samples were used in further analyses. DEGs were identified by comparisons using linear models for microarray data (LIMMA) available in the Bioconductor package [43]. A total of 1,295 DEGs were selected that satisfied the two following criteria, |SLR|>1 and adj. *p*<0.01 (SLR, signal log_2_ ratio; adj. *p*, false discovery rate adjusted *p* value), in at least one comparison for each Affymetrix probe set. The DEGs were further analyzed using fuzzy *k*-means clustering with FANNY function. Clustering results were visualized using the Multi Expression Viewer (MEV) software (http://www.tm4.org/mev/) [44]. Gene ontology (GO) enrichment was evaluated for each cluster with the GO annotation file of *A. thaliana* from http://geneontology.org (downloaded 17 Jan 2012).

### ChIP-seq and ChIP-qPCR Analysis

ChIP-seq libraries were prepared using the protocol of [45] with modifications. Arabidopsis seedlings were grown for 7 days on solid MS medium and hypoxia stressed for 2 hours as described above. Immediately at the termination of treatment, 1 g of plant material was transferred to a 50 ml Falcon tube and fixed in 25 ml MC buffer (10 mM sodium phosphate, pH 7, 50 mM NaCl, 0.1 M sucrose) containing 1% (w/v) formaldehyde by incubation on ice for 20 min. The fixation was stopped with the addition of 2.5 mL of 1.25 M glycine. After three washes with 25 mL MC buffer, the seedlings were frozen, ground and hydrated in 25 mL M1 buffer [10 mM sodium phosphate, pH 7, 0.1 M NaCl, 1 M 2-methyl 2,4-pentanediol, 10 mM β-mercaptoethanol and 0.5 tablet Complete Protease Inhibitor Cocktail (Roche Molecular Diagnostics, Pleasanton, CA) per 25 ml]. The slurry was filtered and centrifuged at 1000 *g* for 20 min at 4°C to obtain a nuclear pellet that was washed five times with 5 ml of M2 buffer (10 mM sodium phosphate, pH 7, 0.1 M NaCl, 10 mM MgCl_2_, 1 M 2-methyl 2,4-pentanediol, 10 mM β-mercaptoethanol and 0.5% (v/v) Triton X-100, 0.5 tablet Complete Protease Inhibitor Cocktail per 25 ml). The final wash was performed with 5 ml M3 buffer (10 mM sodium phosphate, pH 7, 0.1 M NaCl, 10 mM β-mercaptoethanol and 0.5 tablet of Complete Protease Inhibitor Cocktail per 25 ml). The nuclear pellet was resuspended in 1 ml sonication buffer (10 mM sodium phosphate, pH 7, 0.1 M NaCl, 0.5% Sarkosyl, 10 mM EDTA) and sonicated using a Bioruptor UCD-200 (Denville, NJ) on ice, following the manufacturer's instruction. The sample was centrifuged twice at 15600 *g* for 10 min at 4°C and the supernatant was used for chromatin immunoprecipitation (ChIP) with 20 µl of EZview Red ANTI-FLAG® M2 Affinity Gel (Sigma-Aldrich, St. Louis, MO) following the manufacturer's instruction with IP buffer (50 mM HEPES, pH 7.5, 150 mM NaCl, 5 mM MgCl_2_, 10 µM ZnSO_4_, 1% (v/v) Triton X-100, 0.05% (w/v) SDS). After the samples were reverse-crosslinked,, the DNA was purified using the Qiagen PCR purification kit. Library construction involving end repair, A-tailing, and ligation to an adapter was conducted using the End-It DNA End-Repair Kit (Epicentre, Madison, WI), Klenow fragment (New England Biolabs, Ipswich, MA) and Fast-Link DNA Ligation Kit (Epicentre). Two custom barcodes of four nucleotides were used for multiplexing (Col-0 [5′-GTAT-3′] and *OE-HRA1#1* [5′-ACGT-3′]). Samples were submitted to the IIGB Genomics Core Facility, University of California, Riverside, for single-end sequencing with 100 cycles using the Illumina Hiseq2000 platform. Raw data in fastq file format were imported into R using the ShortRead package [46], the barcode sequence was removed from the 100 bp read sequences and reads were aligned to the *A. thaliana* genome TAIR10 version (http://www.arabidopsis.org) using Bowtie (ver. 0.12.7) [47], allowing two nucleotide mismatches. Peaks were generated with the Model-based Analysis for ChIP-Seq (MACS) software [48] using Col-0 mock ChIP data as the control with default settings. ChIPpeakAnno was used to acquire gene annotation, determine location of peak regions from the nearest genes, and obtain DNA sequences of peak regions [49]. Peaks of ChIP-seq data were visualized using the Integrative Genomics Viewer software (2.1) [50].

### SDS-PAGE and Western Blotting

Soluble protein samples from total tissue extracts were separated by SDS-PAGE on 10% polyacrylamide Bis-Tris NuPAGE midigels (Life Technologies) and then transferred onto a polyvinylidene difluoride membrane by means of the Trans-Blot Turbo System (Bio-Rad). Detection of the HRP-conjugated secondary antibody (goat anti-rabbit IgG, Agrisera, product code AS09 602) was performed with the LiteAblot Turbo Extra-Sensitive Chemiluminescent Substrate (EuroClone). Antibodies against PDC (product code AS10 691) and ADH (product code AS10 685) were purchased from Agrisera and antisera against FLAG (A8592) from Sigma-Aldrich. Equal loading of total protein samples was checked by amido black staining, as described in [19].

### Measurement of ADH Activity and Soluble Carbohydrate Levels

ADH-specific activity was measured as described previously [51] with minor modifications, using Arabidopsis 7-d-old seedlings.

Soluble carbohydrates analyzed in Figure S7 were extracted from whole rosettes using perchloric acid and analyzed enzymatically in the neutralized supernatant, as described previously by [52].

### Yeast-Two-Hybrid Assays

The ProQuest Two-Hybrid System (Life Technologies) was used. *Saccharomyces cerevisiae* strain MaV203 was transformed with the different combinations of bait (obtained after recombination of the inserts into pDEST32), prey (obtained after recombination of the inserts into pDEST22), and control vectors. Empty pDEST32 and pDEST22 were used as negative controls. Yeast transformation was performed according to the LiAc/SS carrier DNA/PEG method [53]. After transformation, yeast containing both vectors was grown for 3 d at 28°C on minimal selective dropout medium lacking Leu and Trp (SC-LW medium) to select colonies containing two vectors. Plating was then replicated on selective dropout medium (SC-LWH+3AT medium) lacking Leu, Trp, and His, supplemented with 10 mM 3-aminotriazole (3AT), in order to select colonies containing interacting partners. The strength of the interaction was further verified by β-galactosidase staining (LacZ) following the manufacturer's instructions.

### Statistical Analysis

Significant variations between genotypes or treatments were evaluated statistically by Student's *t* test or one-way ANOVA, coupled with Tukey's posttest, for general comparisons, or Dunnet's posttest, for multiple comparisons with a reference sample, where appropriate. Mean values that were significantly different (*p*<0.05) from each other are marked with lower case letters or asterisks inside the figures. The statistical evaluation of the submergence survival, DNA microarray, and ChIP-seq experiments is described under the respective subsections.

### Phylogenetic Analysis and Analysis of Hypoxia-Inducible Trihelix Genes

Full protein coding sequences of plant trihelix proteins were obtained from GenBank and aligned using ClustalW [54]. The maximum likelihood algorithm in the MEGA5.0 framework [55] was used with 500 bootstrap replicates to evaluate evolutionary relatedness. To identify trihelix genes positively regulated by low oxygen conditions across plant species (Table S1), existing transcriptomic data were surveyed. Genes belonging to the trihelix family in each of the organisms taken into consideration were extracted from the PlantTFDB [56] and used to query the selected public microarray datasets.

## Supporting Information

Figure S1 *HRA1* is the only low oxygen-responsive trihelix-coding gene in Arabidopsis. A comprehensive selection of low oxygen-related Genevestigator [57] datasets generated in *A. thaliana* (63 experiments) was queried for the expression of all the trihelix genes encoded in the genome (30 genes, of which 26 of the corresponding Affymetrix probes were found) and then filtered on *At3g10040* (*HRA1*), according to the following criteria: |Fold change|≥2, *p*<0.05.
(TIF)

Figure S2 HRA1 is a member of the plant trihelix TF family. Sequences of the conserved trihelix domain (69 amino acids in GT-1) of Arabidopsis, rice, and poplar trihelix protein family members were used for phylogenetic tree reconstruction as described previously [14]. Alignment of the sequences was performed using ClustalW [54]. A phylogenetic tree was produced by use of the maximum likelihood algorithm with 1000 bootstrap replications using the MEGA5 software [55]. The scale bar below the phylogenetic tree indicates 0.5 amino acid substitutions per site. Values represent bootstrap frequency (>50%). Labeling of trihelix clades is from [14]. The protein SAB18 (Os11g06410), clustering with GT-γ trihelix factors, was reported to interact with SUB1A and SUB1C of rice (*Oryza sativa*) in a yeast-two-hybrid assay and bimolecular fluorescence complementation [23]. *SH4-like2* (At1g31310) was identified as a putative target of HRA1 by ChIP-seq analysis (Table S3). Low oxygen-inducible trihelix proteins of poplar and soybean in Table S1 are also shown in the phylogenetic tree (GTγ clade, pmrna35920, Glyma13g21350, and Glyma19g37410; GT-1 clade, Glyma01g29760; GT-2 clade, pmrna11072, CX177654, and Glyma06g15500; SIP1 clade, pmrna37656). Rice gene names correspond to the Michigan State University Rice Genome (Osa1) Annotation Release 7.
(TIF)

Figure S3 *HRA1* is an early hypoxia-responsive gene. (A) *HRA1* mRNA is kept constant at medium to low levels across Arabidopsis tissues at various plant developmental stages. Average gene expression data were retrieved from the Genevestigator webtool [57] on August 1, 2013. (B) *HRA1* mRNA accumulation is not sustained along the progression of the stress, unlike the case of the typical hypoxia marker gene *ADH1*. Data are mean ± s.d. (*n* = 3) from RT-qPCR analyses. Aerial and root tissues were collected from 3-wk-old plants grown on solid MS medium. Aerobic shoot samples were used as the reference. (C) The hypoxia-induced *HRA1* mRNA is translated ubiquitously in the plant. The cartoons depict the absolute signal values of *HRA1* transcript in translatomes (polysome-associated mRNA populations) isolated from different leaf and root cell types [15], as visualized by the eFP platform available at www.efp.ucr.edu.
(TIF)

Figure S4 Molecular features of HRA1 overexpressing and mutant lines used in this study. (A) Western blot showing the accumulation of the HRA1-FLAG protein in *OE-HRA1#1* and *OE-HRA1#2* 7-d-old seedlings (*35S:HRA1:FLAG* genotype), in aerobic conditions, after 2 h hypoxia and after subsequent recovery in normal atmosphere (2 h). (B) Schematic diagram of the *HRA1* open reading frame (light grey, untranslated sequences; blue, coding sequence). Triangles mark the positions of the two T-DNAs present in the insertional mutants *hra1-1* (red triangle) and *hra1-2* (orange triangle). Occurrence of tandem T-DNA repetitions could be inferred after sequencing of their flanking regions (see Figure S5 for more details), upon recovery of left border sequences on both T-DNA extremities, but the number of repetitions in each mutant remains undetermined. Finally, the light green and light blue segments under the scheme correspond to the HRA1_Tot_ and HRA1_Endo_ RT-qPCR amplicons shown in Figure 5B. (C) RT-PCR, followed by gel electrophoresis, showing *HRA1* transcripts level in the two *hra1* mutants and the wild type. The mRNA was isolated from 7-d-old seedlings exposed to control or hypoxic conditions (2 h hypoxia in the dark), and 30 cycles of amplification were performed, using primer set1 and 2 (see Table S8). The analysis confirms that both the insertion alleles failed to produce a complete gene transcript.
(TIF)

Figure S5 Sequencing of the T-DNA flanking regions in the *hra1-1* and *hra1-2* mutant alleles. For the amplification of either *hra1-1* or *hra1-2* genomic DNA, the LBb1 primer, annealing on the left T-DNA border, was used in combination with an upstream (gwHRA1_580_Fw) or downstream (gwHRA1_Rv) primer (see Table S8). The PCR products obtained were cloned in the pGEM®-T Easy vector (Promega) and analyzed by Sanger sequencing with an SP6-specific primer. The output of the four sequencing reactions is displayed: Primer sequences are underlined, and upper- and lower-case letters mark the parts corresponding to *HRA1* genomic sequence and T-DNA left border, respectively. Insertion of the T-DNAs was accompanied by ablation of nucleotides 1335–1355 from the gene, in the *hra1-1* allele, and nucleotides 1433–1465 in *hra1-2*.
(TIF)

Figure S6 Altered HRA1 levels reduce plant ability to survive submergence. (A) Representative pictures (upper part) and median lethal time (LT50; lower part) of wild type, *hra1* mutant, and *HRA1* overexpressing plants at the 10-leaf rosette stage (stage 1.10 [30]), after complete submergence in the dark. Photographs were taken at the end of the treatment, before the recovery period. Plants were treated and scored exactly as described in [16]. Submergence tolerance data of hypoxia-responsive unknown protein (HUP) mutants reported previously [16] are included in the LT50 graph, to facilitate the comparison between *HRA1*, formerly referred to as *HUP14*, with other known hypoxia-related genotypes. The standard deviation of Col-0 was obtained from multiple datasets. Red and green indicate a statistically significant difference between the selected *HRA1* genotypes and the wild type (95% confidence interval values). Slight differences in tolerance were detected between plants treated at this developmental age and older plants (compare to results Figure 3A and B). (B) Petiole elongation is reduced by *HRA1* overexpression in air but not as a response to submergence. Petiole lengths were recorded at the 10-leaf rosette stage (L0) and after 3 d of treatments (air control and submergence; L3), and petiole elongation rates were calculated as (L3−L0)/3. Data are mean ± s.d. (*n* = 10).
(TIF)

Figure S7 Soluble sugar contents in soil-grown plants at the late vegetative rosette stage (3.90) of development. The amount of soluble sugars (glucose, fructose, sucrose, and their sum) was assessed, at the beginning (ZT0, 8 a.m.) and the end of the photoperiod (ZT12, 8 p.m.), in the aerial tissues of plants of the same age as those evaluated in Figure 3A for submergence tolerance. Soluble carbohydrates were extracted from whole rosettes (*n* = 3). Data are mean ± s.d. One-way ANOVA on single time points was performed, and no statistically significant differences from the wild type were found.
(TIF)

Figure S8 Patterns of *HRA1* and *ADH1* expression over midterm submergence. *HRA1* responds to submergence-induced hypoxia and peaked within the first 6 h, in the wild type. *ADH1* was measured as a downstream target of the treatment and equally produced a peak of expression within the first 6 h of treatment, although the reciprocal position of *ADH1* and *HRA1* expression maxima cannot be concluded from the present analysis. Data are mean ± s.d. (*n* = 3) of relative mRNA levels, normalized by setting as 1 the wild type expression value in air (time = 0) for both the old and the young leaves. Note the higher steady-state level mRNA of both hypoxia markers in the young leaves, either under aerated conditions and over the stress. Asterisks mark statistically significant differences, in the overexpressors (blue asterisks) or in the mutant (red asterisks), from the wild type mean expression value after separate one-way ANOVA for each time point (*p*<0.05). Differences in *ADH1* expression in the *hra1-1* mutant, which are present at 2 and 6 h submergence in the young leaves only and disappear towards the end of the treatment, are supposed to be correlated with the time span of HRA1 activity.
(TIF)

Figure S9 Young leaves of *hra1-1* and *hra1-2* plants display a similar molecular phenotype. Comparison of *ADH1*, *PDC1*, and *HRA1* mRNA levels in young leaf samples from 4-wk-old air-grown plants indicates that basal expression of hypoxia markers is higher than the wild type in either *hra1-1* (see also Figure 3C) or the independent *hra1-2* mutant. Data related to the *OE-HRA1#1* genotype are included for comparison. Each biological sample was obtained by the collection of young leaf tissue from at least five plants. Data are mean ± s.d. (*n* = 3) of relative mRNA levels, normalized by setting the wild type expression value as 1. Asterisks mark statistically significant differences from the wild type after one-way ANOVA (*p*<0.05, Dunnet's posttest).
(TIF)

Figure S10 Images of the Western blot membranes magnified in Figure 3D. PDC and ADH were immunodetected in wild type, *OE-HRA1#1*, and *hra1-1* leaf samples using specific polyclonal antibodies, as indicated by the arrows. The same membrane was cut in two parts for separate hybridization with the different antisera, and each portion was eventually exposed for the convenient time for protein detection. We loaded 50 µg total proteins in each well, and equal loading of the samples was visualized through amido black staining of the PVDF membrane [19]. The blot shown is representative of three biological replicates.
(TIF)

Figure S11 Profile of *HRA1* promoter activity in 4-wk-old plants, visualized by GUS-reporter staining. During normal growth (“Control”), the promoter was active in the apical meristem zone, which includes the emerging leaves, and in leaf veins. After 12 h of submergence, the activity was markedly enhanced in the same tissues but also expanded to mesophyll and epidermal tissues. Scale bar, 1 cm.
(TIF)

Figure S12 Excessive HRA1 accumulation is detrimental for survival to mild (2 d) or prolonged (9 d) continuous darkness. Mature leaves from *OE-HRA1* plants are more strongly affected by carbon starvation and senesce earlier than the other genotypes. Plants were grown as in [16] before being shifted to continuous darkness.
(TIF)

Figure S13 Phenotypical characterization of *HRA1* overexpressing plants. (A) Representative pictures of wild type, *HRA1* mutant, and overexpressing plants, grown at the end of the vegetative phase (stage 3.90 [30], here corresponding to 26 d of growth in soil under a 12 h light∶12 h darkness neutral photoperiod). Scale bar, 3 cm. *OE-HRA1#1* and *OE-HRA1#2* are *35:HRA1:FLAG* overexpressors, and *OE-HRA1#3* indicates, instead, an independent *35S:HRA1* transgenic line. Note the shortened petiole and reduced leaf index (ratio between length and width) in the overexpressing plants. No obvious differences from the wild type were recorded in the *hra1-1* or *hra1-2* mutants. (B) Rosette growth parameters and total leaf anthocyanin content in *OE-HRA1* soil-grown plants at a stage corresponding to stage 3.90 in the wild type [30]. Overexpressing plants are more compact (reduced petiole elongation and lower rosette max diameter) and produce a higher number of leaves, which however are smaller than in the wild type (lower total fresh weight). Data are mean ± s.d. (*n* = 4 for rosette FW and anthocyanin content; *n* = 6 for rosette size and leaf number). *p<0.05 statistically significant means from the wild type, after one-way ANOVA. (C) Above, phenotype of plants progressing through the reproductive phase (principal stage 4 [30]; 46-d-old plants were photographed here). A developmental delay and partial loss of apical dominance was highlighted in the *OE-HRA1* genotypes, whereas *HRA1* mutants did not display significant alterations. Flowering time of *OE-HRA1#1* plants (bottom left) was assessed both in terms of days from germination and number of leaves to floral induction and consistently showed a delay in the transgenic plants if compared with the wild type, independent of the photoperiodic flowering pathway (LD, long day, 15 h light∶9 h darkness; ND, neutral day). Mean ± s.d. (*n* = 12), *p<0.05 statistically significant means from the wild type, after one-way ANOVA. Data describing plant yield (bottom right) are mean ± s.d. (*n* = 4). *p<0.05 statistically significant differences from the wild type after one-way ANOVA.
(TIF)

Figure S14 Identification of HRA1 binding sites by chromatin immunopurification and fragment sequencing (ChIP-seq). (A) Pie chart displaying the percentage of HRA1 binding peaks that map to defined domains of annotated genes. Upstream, peak maps 5′ of the predicted transcription start site; downstream, peak maps 3′ of the transcriptional unit of the gene; inside, peak maps within the annotated mRNA; overlapStart, peak overlaps with the predicted transcriptional start site of the gene; overlapEnd, peak overlaps with the end of the gene; includeFeature, peak includes the entire gene. (B) Venn diagram comparison of the 1,295 DEGs, identified by five comparisons in DNA microarray analysis, and the 146 putative targets of HRA1 binding, identified by ChIP-Seq. The overlap was seven genes. (C) Heatmap summarizing the expression values of the seven genes identified in both the DEG and ChIP-Seq datasets (Figure S14B), as extracted from the DNA microarray datasets.
(TIF)

Figure S15 HRA1 interacts with RAP2.12. (A) Schematic view of the domains present on HRA1 and RAP2.12 proteins. (B) Yeast-two-hybrid between an N-terminal RAP2.12 fragment (bait) and HRA1 (prey), showing that amino acids 1–123 of RAP2.12, corresponding to a variable region preceding the ERF DNA binding domain [7], are sufficient for the interaction. (C) An N-terminal HRA1 deletion version (HRA1_194–431_), lacking the predicted trihelix DNA binding domain, is able to interact with RAP2.12 (RAP2.12_1–177_) in the yeast-two-hybrid assay and also when used as the prey construct.
(TIF)

Figure S16 Interaction of HRA1 with RAP2.12 has an impact *in vivo*. (A) Survival of protoplasts incubated for 18 h was evaluated by calculating the proportion of alive, fluorescein diacetate-stained, cells [58] per unit volume on a hemocytometer under a fluorescence microscope. Incubation of protoplasts was static and in darkness, making the occurrence of hypoxia in the cells very likely. Data are mean ± s.d. (*n* = 8), and *p<0.05 indicates statistically significant difference from the control transformation (first column, protoplasts transformed with *35S:RrLuc* only). Protoplasts were transfected with 3 µg individual plasmid DNA in every transformation. (B) Phenotype of soil-grown plants overexpressing HRA1 alone or HRA1 and the stable RAP2.12_14–358_ version at the same time.
(TIF)

Table S1 List of low oxygen-inducible trihelix coding genes in four mono- and dicotyledonous plant species. In bold are genes belonging to the same orthologous group as *HRA1* according to the PlantTFDB database.
(DOCX)

Table S2 DNA microarray analysis of *OE-HRA1* lines.
(XLSX)

Table S3 Chromatin immunopurification and fragment sequencing analysis with *OE-HRA1#1*. The *p* values indicate significance of fold changes based on Poisson distribution.
(XLSX)

Table S4 List of ChIP-PCR primers.
(DOCX)

Table S5 Comparison of total *HRA1*, endogenous *HRA1* and *ADH1* transcript levels in wild type, and *OE-HRA1* and *hra1* seedlings in normoxia or after 2 h hypoxia. Fold change values were calculated relatively to the wild type control. Data are means ± s.d. (*n = 4*). *p adj. <0.05 and **p adj. <0.01 were calculated separately for control and hypoxic conditions, after one-way-ANOVA.
(DOCX)

Table S6 Referenced list of the plasmid constructs produced in this study.
(DOCX)

Table S7 Oligonucleotide primers used for gene cloning and RT-PCR screening.
(DOCX)

Table S8 Oligonucleotide primers used for qPCR analyses.
(DOCX)

## Abbreviations

ADH: alcohol dehydrogenase
ERF-VII: ethylene-responsive factor group VII
GFP: green fluorescent protein
GUS: beta-glucoronidase
HRA1: hypoxia response attenuator 1
HREs: hypoxia responsive ERFs
PDC: pyruvate decarboxylase
RAP2.12: related to AP2 12
TF: transcription factor
UAS: upstream activating sequence of the yeast *GAL4* gene
