# Supplementary figures and images for: A Trihelix DNA Binding Protein Counterbalances Hypoxia-Responsive Transcriptional Activation in Arabidopsis
Source: PLoS Biol. 2014 Sep 16;12(9):e1001950. doi: 10.1371/journal.pbio.1001950 (PMC4165759; doi:10.1371/journal.pbio.1001950)

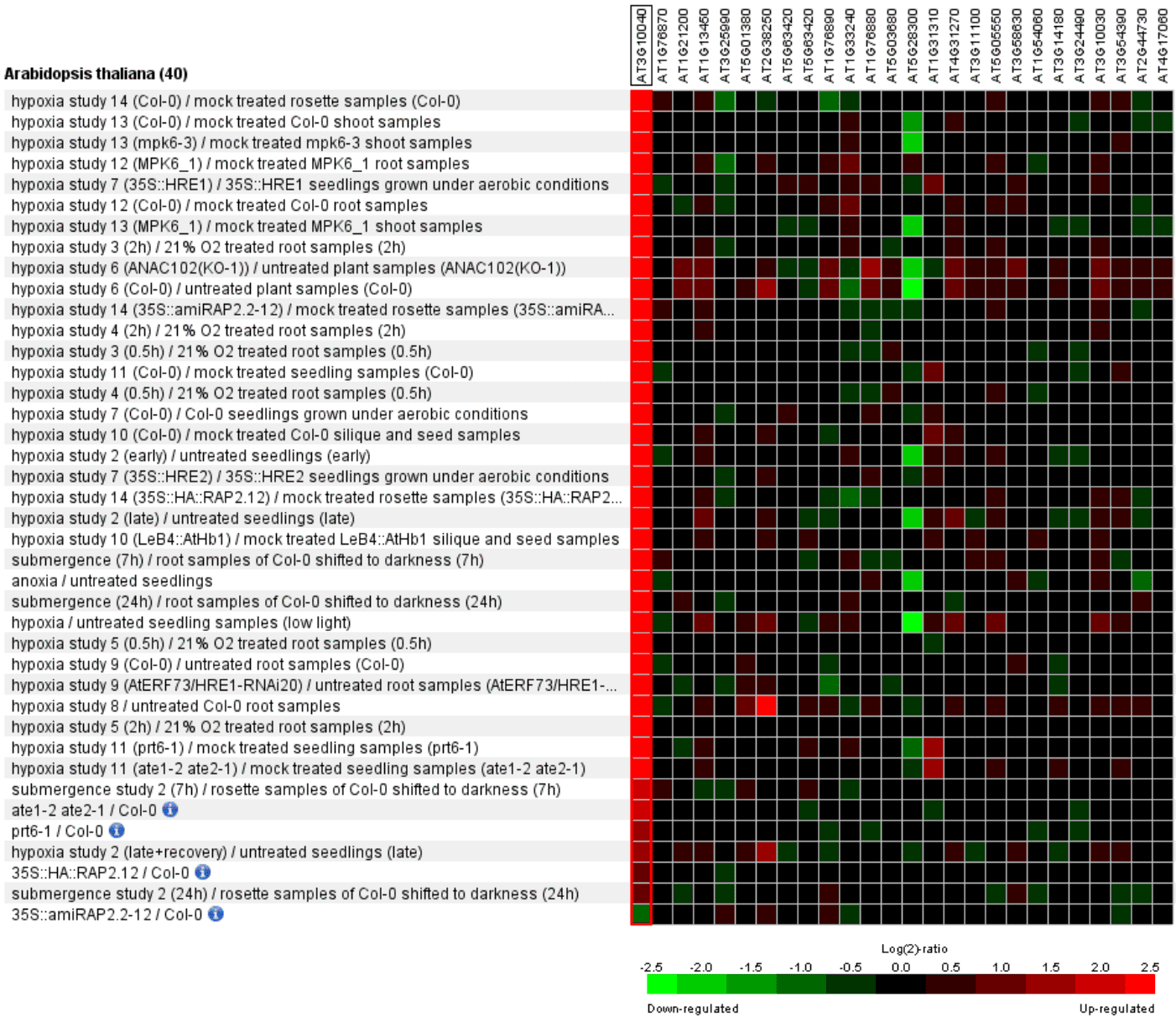

Supplement: Figure S1 — HRA1 is the only low oxygen-responsive trihelix-coding gene in Arabidopsis. A comprehensive selection of low oxygen-related Genevestigator [57] datasets generated in A. thaliana (63 experiments) was queried for the expression of all the trihelix genes encoded in the genome (30 genes, of which 26 of the corresponding Affymetrix probes were found) and then filtered on At3g10040 (HRA1), according to the following criteria: |Fold change|≥2, p<0.05. (TIF) [file pbio.1001950.s001.tif]

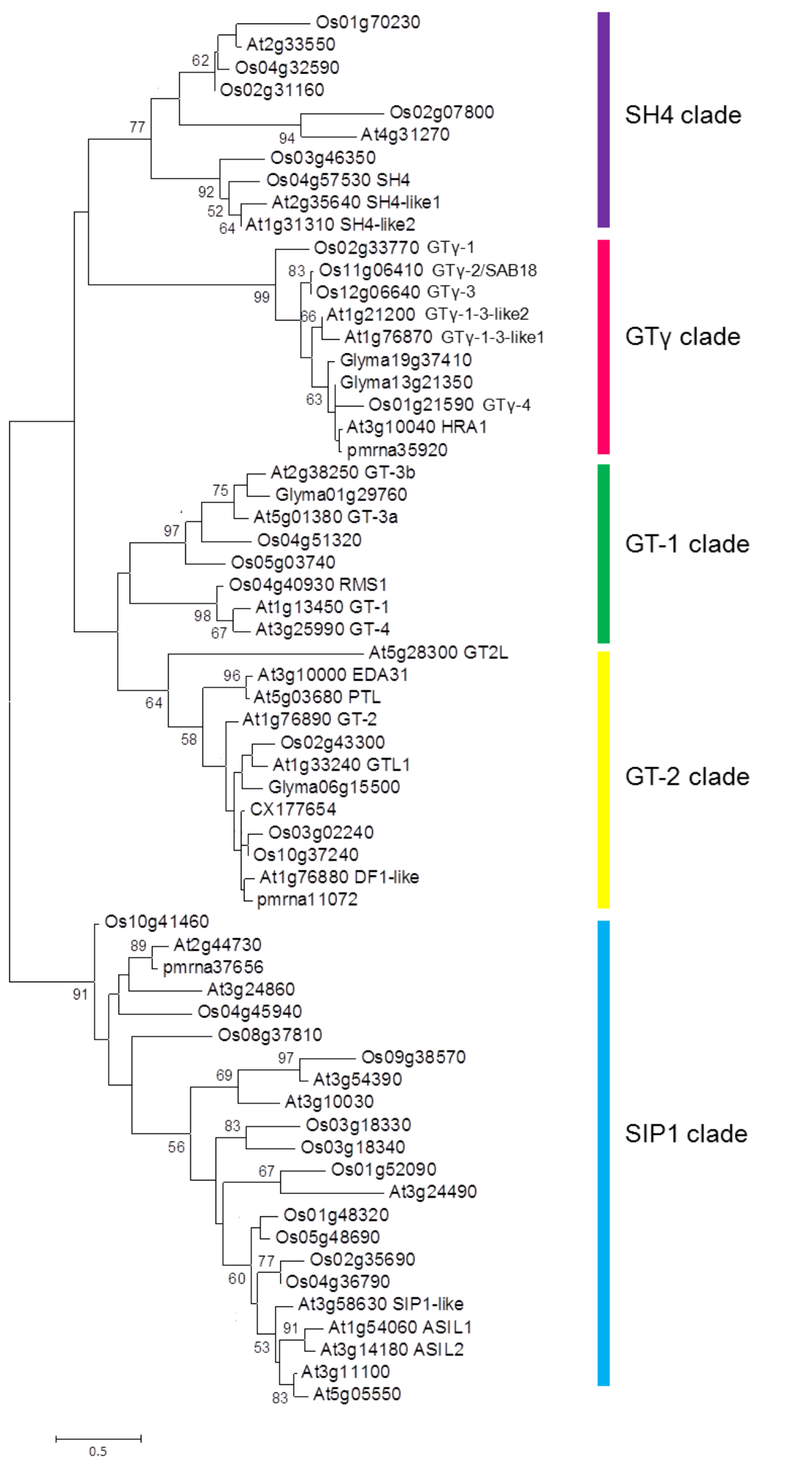

Supplement: Figure S2 — HRA1 is a member of the plant trihelix TF family. Sequences of the conserved trihelix domain (69 amino acids in GT-1) of Arabidopsis, rice, and poplar trihelix protein family members were used for phylogenetic tree reconstruction as described previously [14]. Alignment of the sequences was performed using ClustalW [54]. A phylogenetic tree was produced by use of the maximum likelihood algorithm with 1000 bootstrap replications using the MEGA5 software [55]. The scale bar below the phylogenetic tree indicates 0.5 amino acid substitutions per site. Values represent bootstrap frequency (>50%). Labeling of trihelix clades is from [14]. The protein SAB18 (Os11g06410), clustering with GT-γ trihelix factors, was reported to interact with SUB1A and SUB1C of rice (Oryza sativa) in a yeast-two-hybrid assay and bimolecular fluorescence complementation [23]. SH4-like2 (At1g31310) was identified as a putative target of HRA1 by ChIP-seq analysis (Table S3). Low oxygen-inducible trihelix proteins of poplar and soybean in Table S1 are also shown in the phylogenetic tree (GTγ clade, pmrna35920, Glyma13g21350, and Glyma19g37410; GT-1 clade, Glyma01g29760; GT-2 clade, pmrna11072, CX177654, and Glyma06g15500; SIP1 clade, pmrna37656). Rice gene names correspond to the Michigan State University Rice Genome (Osa1) Annotation Release 7. (TIF) [file pbio.1001950.s002.tif]

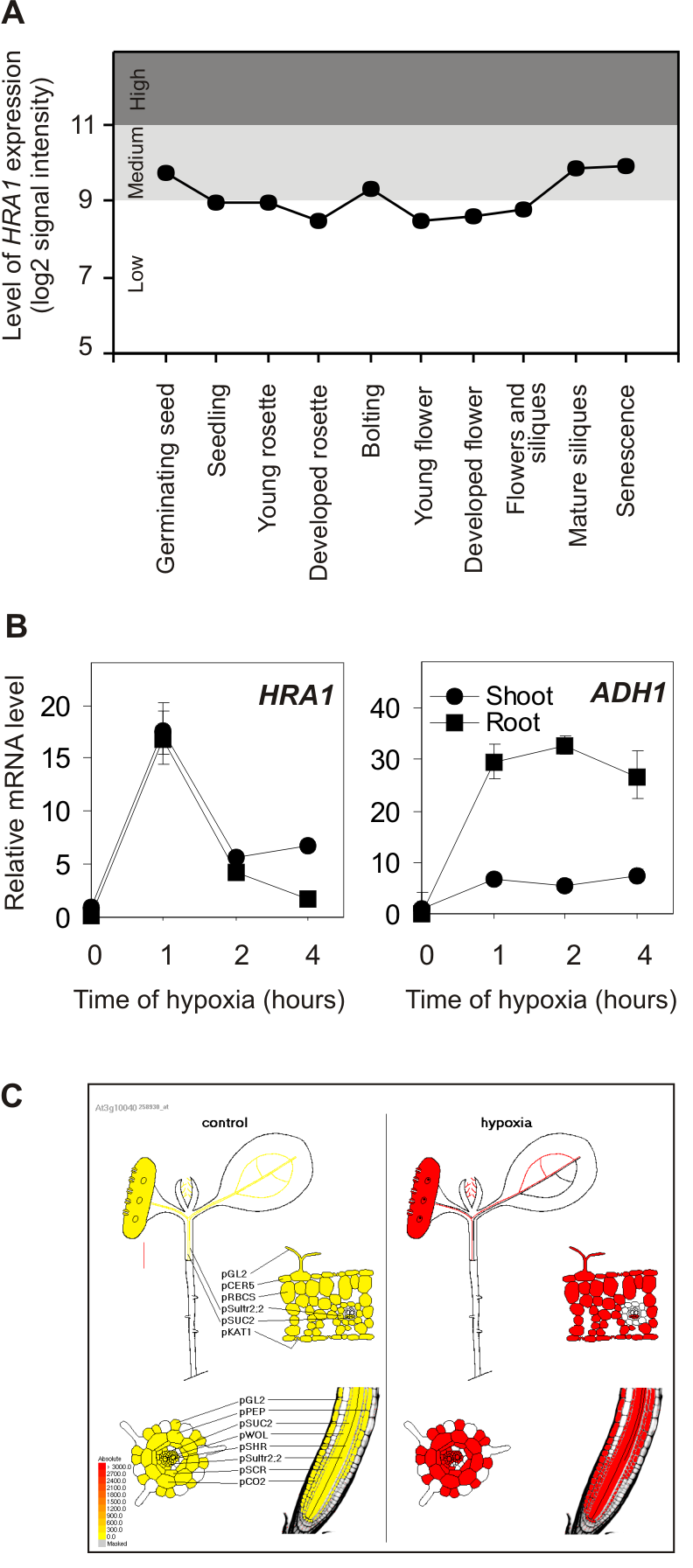

Supplement: Figure S3 — HRA1 is an early hypoxia-responsive gene. (A) HRA1 mRNA is kept constant at medium to low levels across Arabidopsis tissues at various plant developmental stages. Average gene expression data were retrieved from the Genevestigator webtool [57] on August 1, 2013. (B) HRA1 mRNA accumulation is not sustained along the progression of the stress, unlike the case of the typical hypoxia marker gene ADH1. Data are mean ± s.d. (n = 3) from RT-qPCR analyses. Aerial and root tissues were collected from 3-wk-old plants grown on solid MS medium. Aerobic shoot samples were used as the reference. (C) The hypoxia-induced HRA1 mRNA is translated ubiquitously in the plant. The cartoons depict the absolute signal values of HRA1 transcript in translatomes (polysome-associated mRNA populations) isolated from different leaf and root cell types [15], as visualized by the eFP platform available at www.efp.ucr.edu. (TIF) [file pbio.1001950.s003.tif]

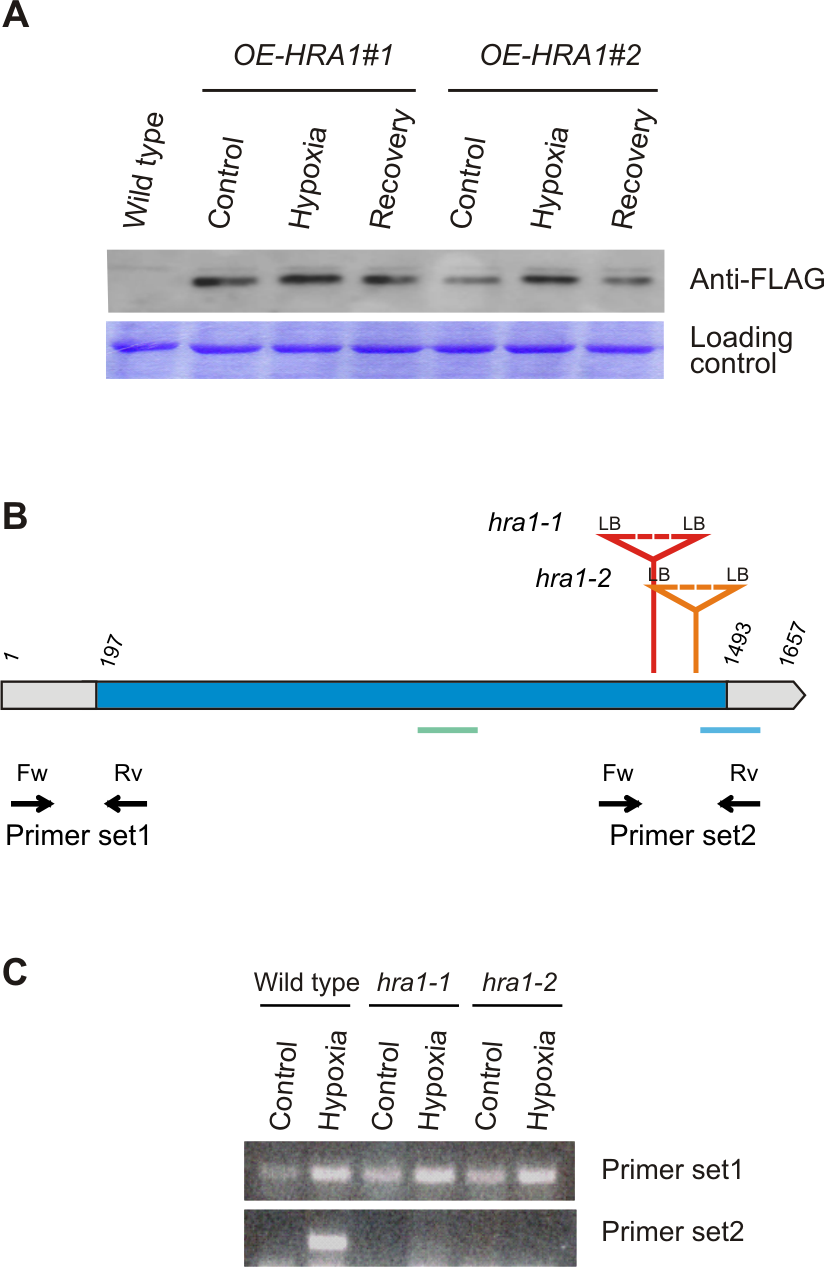

Supplement: Figure S4 — Molecular features of HRA1 overexpressing and mutant lines used in this study. (A) Western blot showing the accumulation of the HRA1-FLAG protein in OE-HRA1#1 and OE-HRA1#2 7-d-old seedlings (35S:HRA1:FLAG genotype), in aerobic conditions, after 2 h hypoxia and after subsequent recovery in normal atmosphere (2 h). (B) Schematic diagram of the HRA1 open reading frame (light grey, untranslated sequences; blue, coding sequence). Triangles mark the positions of the two T-DNAs present in the insertional mutants hra1-1 (red triangle) and hra1-2 (orange triangle). Occurrence of tandem T-DNA repetitions could be inferred after sequencing of their flanking regions (see Figure S5 for more details), upon recovery of left border sequences on both T-DNA extremities, but the number of repetitions in each mutant remains undetermined. Finally, the light green and light blue segments under the scheme correspond to the HRA1Tot and HRA1Endo RT-qPCR amplicons shown in Figure 5B. (C) RT-PCR, followed by gel electrophoresis, showing HRA1 transcripts level in the two hra1 mutants and the wild type. The mRNA was isolated from 7-d-old seedlings exposed to control or hypoxic conditions (2 h hypoxia in the dark), and 30 cycles of amplification were performed, using primer set1 and 2 (see Table S8). The analysis confirms that both the insertion alleles failed to produce a complete gene transcript. (TIF) [file pbio.1001950.s004.tif]

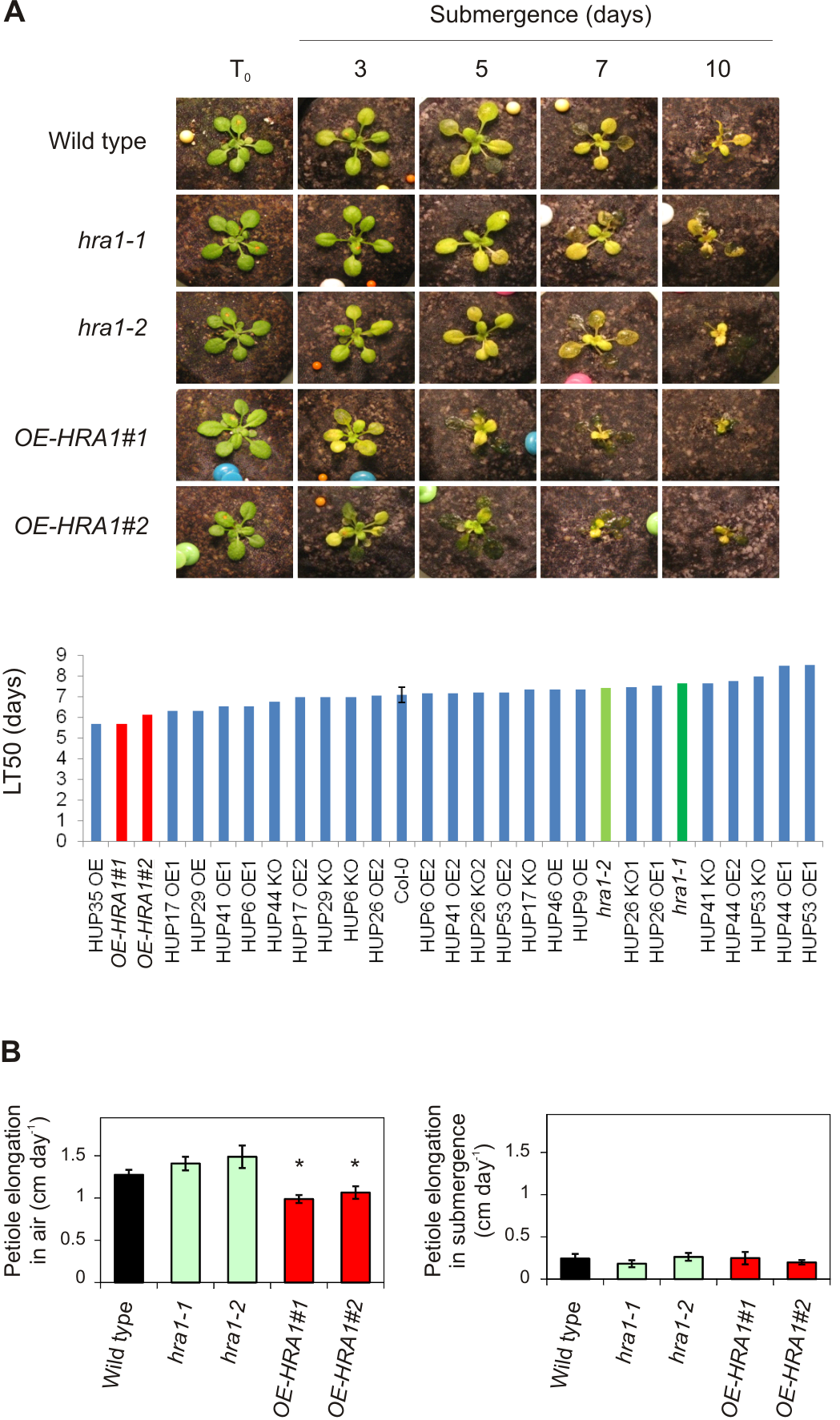

Supplement: Figure S6 — Altered HRA1 levels reduce plant ability to survive submergence. (A) Representative pictures (upper part) and median lethal time (LT50; lower part) of wild type, hra1 mutant, and HRA1 overexpressing plants at the 10-leaf rosette stage (stage 1.10 [30]), after complete submergence in the dark. Photographs were taken at the end of the treatment, before the recovery period. Plants were treated and scored exactly as described in [16]. Submergence tolerance data of hypoxia-responsive unknown protein (HUP) mutants reported previously [16] are included in the LT50 graph, to facilitate the comparison between HRA1, formerly referred to as HUP14, with other known hypoxia-related genotypes. The standard deviation of Col-0 was obtained from multiple datasets. Red and green indicate a statistically significant difference between the selected HRA1 genotypes and the wild type (95% confidence interval values). Slight differences in tolerance were detected between plants treated at this developmental age and older plants (compare to results Figure 3A and B). (B) Petiole elongation is reduced by HRA1 overexpression in air but not as a response to submergence. Petiole lengths were recorded at the 10-leaf rosette stage (L0) and after 3 d of treatments (air control and submergence; L3), and petiole elongation rates were calculated as (L3−L0)/3. Data are mean ± s.d. (n = 10). (TIF) [file pbio.1001950.s006.tif]

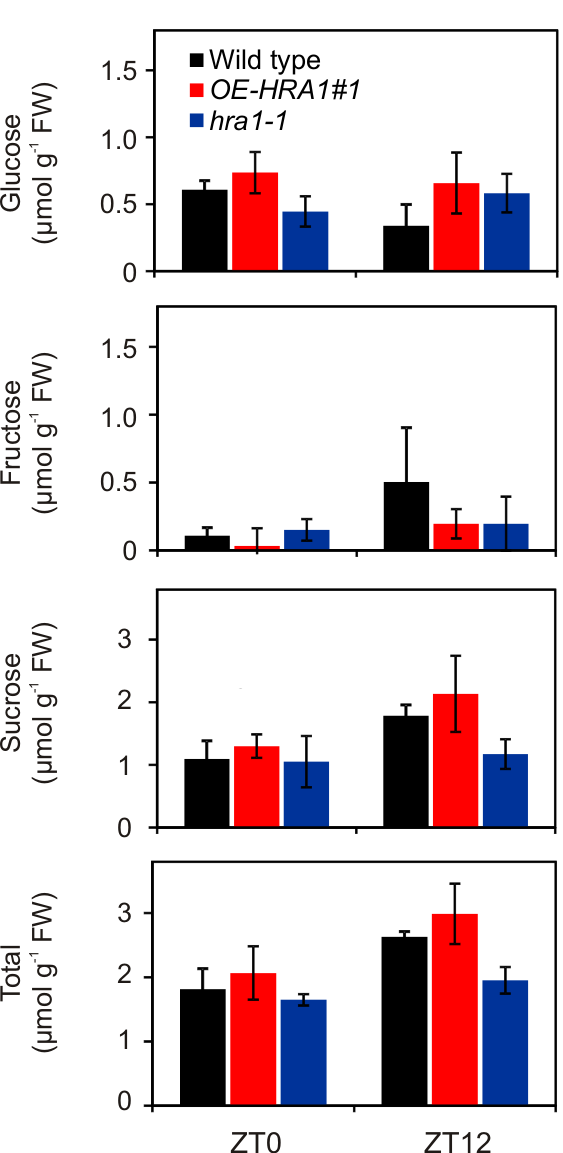

Supplement: Figure S7 — Soluble sugar contents in soil-grown plants at the late vegetative rosette stage (3.90) of development. The amount of soluble sugars (glucose, fructose, sucrose, and their sum) was assessed, at the beginning (ZT0, 8 a.m.) and the end of the photoperiod (ZT12, 8 p.m.), in the aerial tissues of plants of the same age as those evaluated in Figure 3A for submergence tolerance. Soluble carbohydrates were extracted from whole rosettes (n = 3). Data are mean ± s.d. One-way ANOVA on single time points was performed, and no statistically significant differences from the wild type were found. (TIF) [file pbio.1001950.s007.tif]

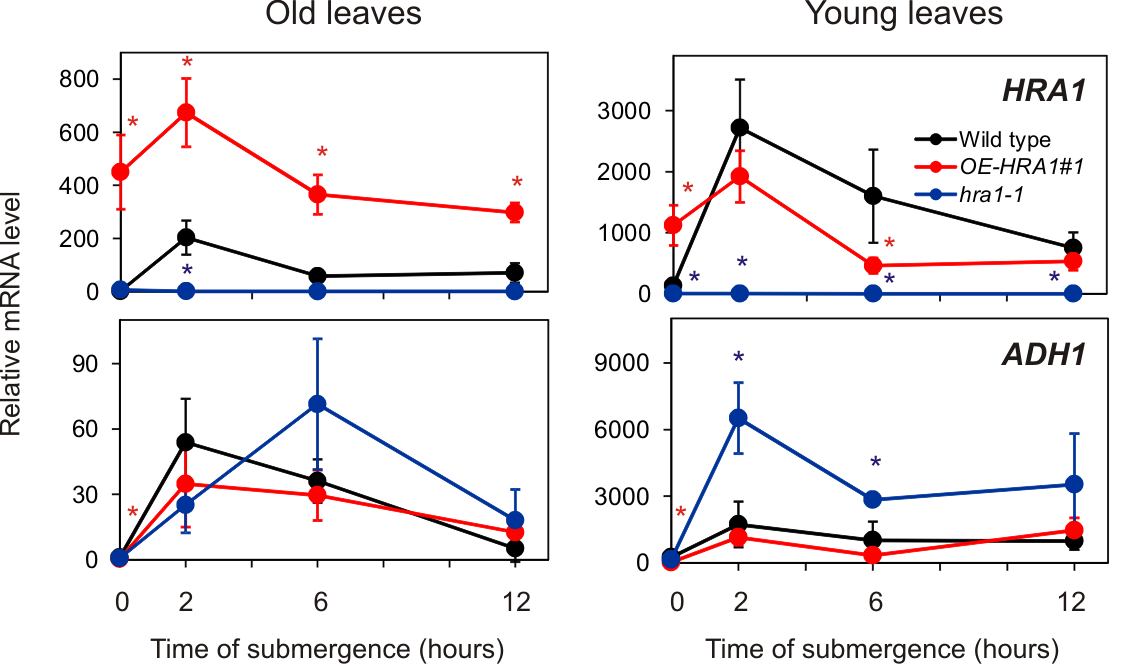

Supplement: Figure S8 — Patterns of HRA1 and ADH1 expression over midterm submergence. HRA1 responds to submergence-induced hypoxia and peaked within the first 6 h, in the wild type. ADH1 was measured as a downstream target of the treatment and equally produced a peak of expression within the first 6 h of treatment, although the reciprocal position of ADH1 and HRA1 expression maxima cannot be concluded from the present analysis. Data are mean ± s.d. (n = 3) of relative mRNA levels, normalized by setting as 1 the wild type expression value in air (time = 0) for both the old and the young leaves. Note the higher steady-state level mRNA of both hypoxia markers in the young leaves, either under aerated conditions and over the stress. Asterisks mark statistically significant differences, in the overexpressors (blue asterisks) or in the mutant (red asterisks), from the wild type mean expression value after separate one-way ANOVA for each time point (p<0.05). Differences in ADH1 expression in the hra1-1 mutant, which are present at 2 and 6 h submergence in the young leaves only and disappear towards the end of the treatment, are supposed to be correlated with the time span of HRA1 activity. (TIF) [file pbio.1001950.s008.tif]

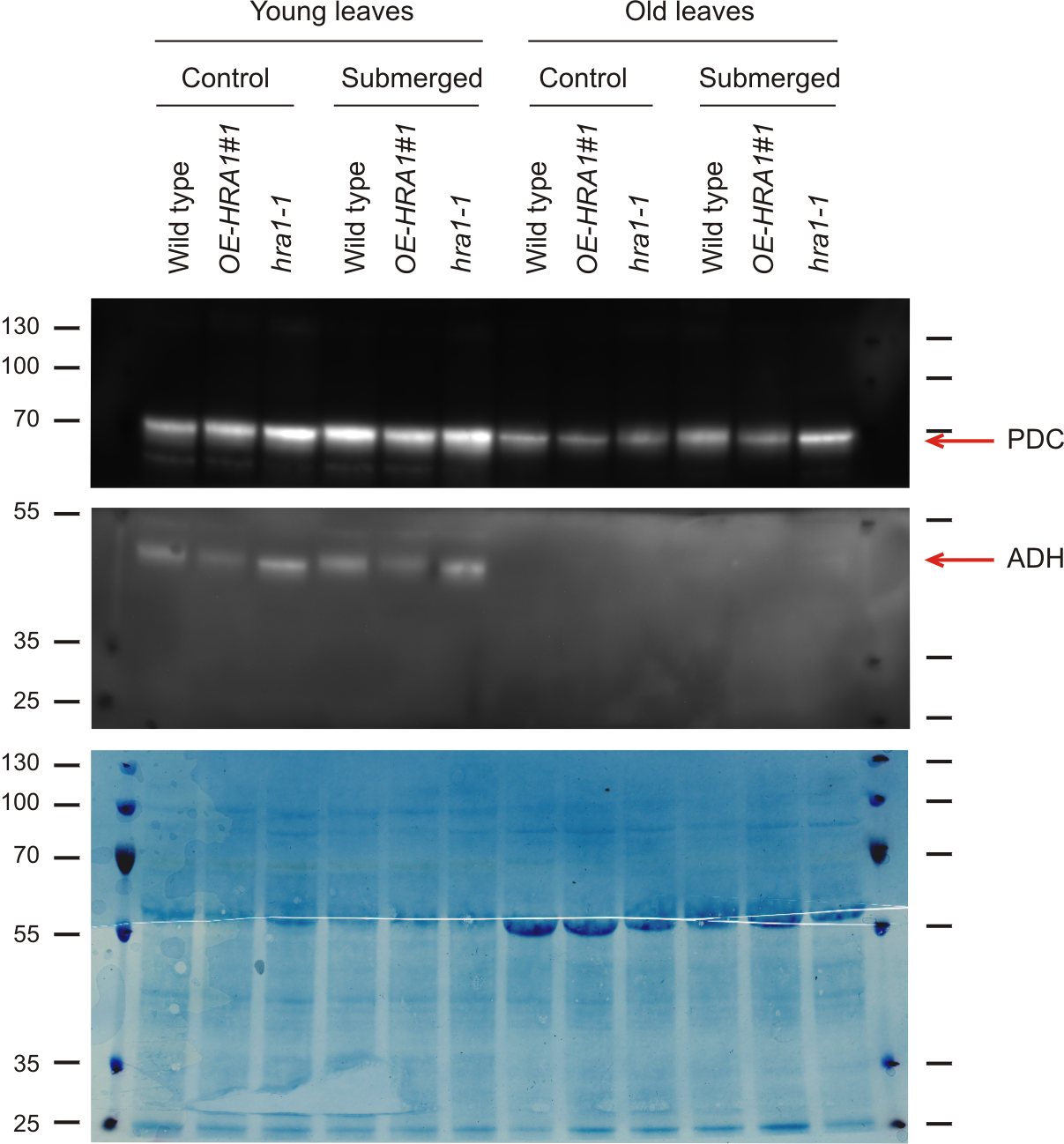

Supplement: Figure S10 — Images of the Western blot membranes magnified in Figure 3D. PDC and ADH were immunodetected in wild type, OE-HRA1#1, and hra1-1 leaf samples using specific polyclonal antibodies, as indicated by the arrows. The same membrane was cut in two parts for separate hybridization with the different antisera, and each portion was eventually exposed for the convenient time for protein detection. We loaded 50 µg total proteins in each well, and equal loading of the samples was visualized through amido black staining of the PVDF membrane [19]. The blot shown is representative of three biological replicates. (TIF) [file pbio.1001950.s010.tif]

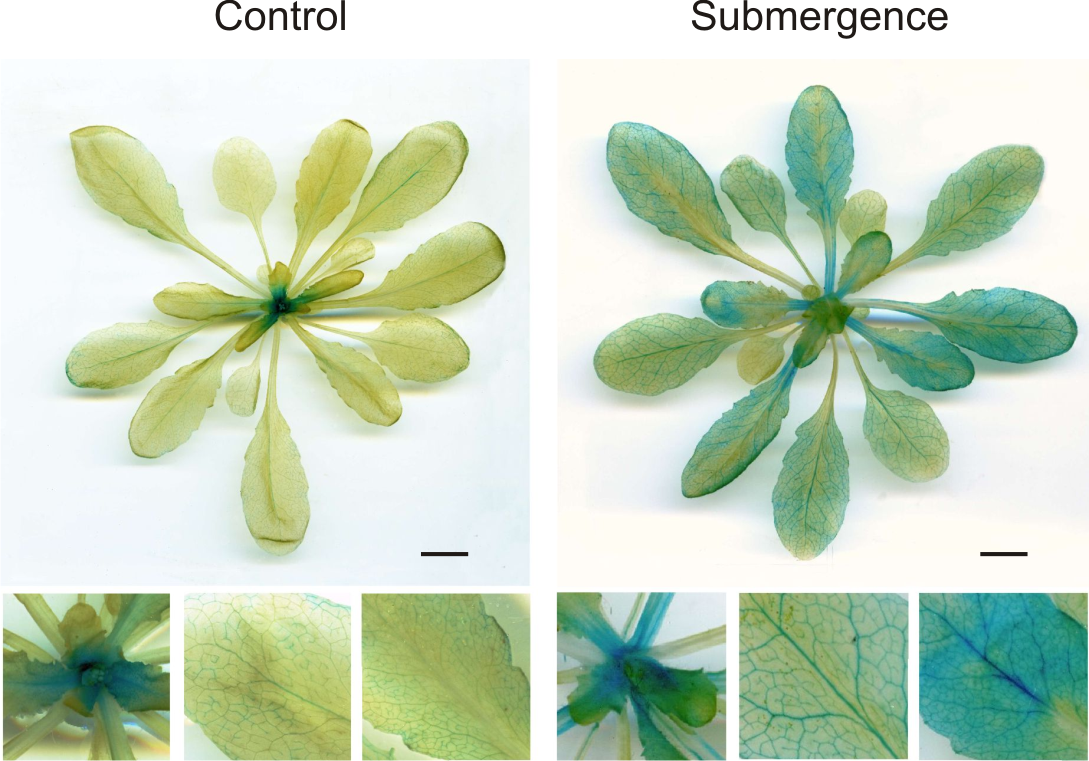

Supplement: Figure S11 — Profile of HRA1 promoter activity in 4-wk-old plants, visualized by GUS-reporter staining. During normal growth (“Control”), the promoter was active in the apical meristem zone, which includes the emerging leaves, and in leaf veins. After 12 h of submergence, the activity was markedly enhanced in the same tissues but also expanded to mesophyll and epidermal tissues. Scale bar, 1 cm. (TIF) [file pbio.1001950.s011.tif]

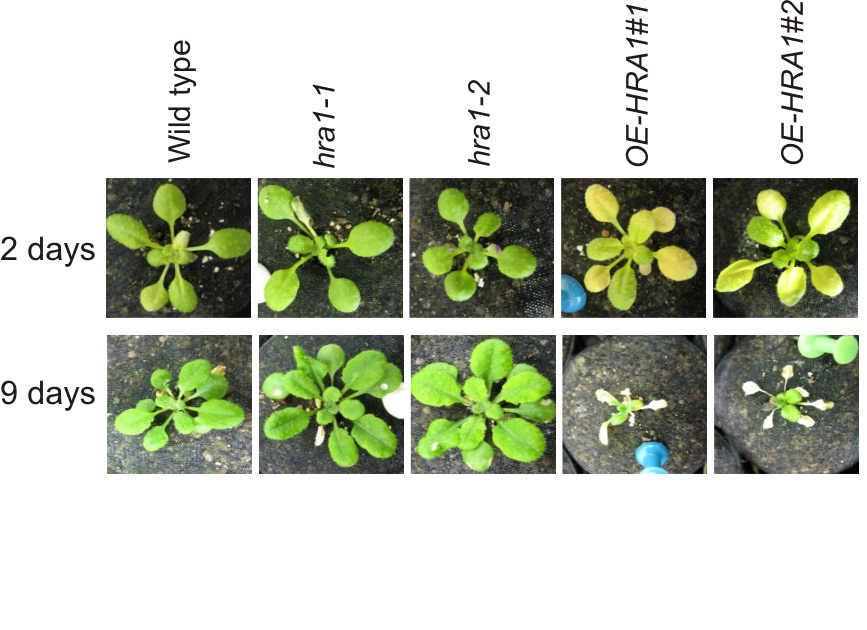

Supplement: Figure S12 — Excessive HRA1 accumulation is detrimental for survival to mild (2 d) or prolonged (9 d) continuous darkness. Mature leaves from OE-HRA1 plants are more strongly affected by carbon starvation and senesce earlier than the other genotypes. Plants were grown as in [16] before being shifted to continuous darkness. (TIF) [file pbio.1001950.s012.tif]

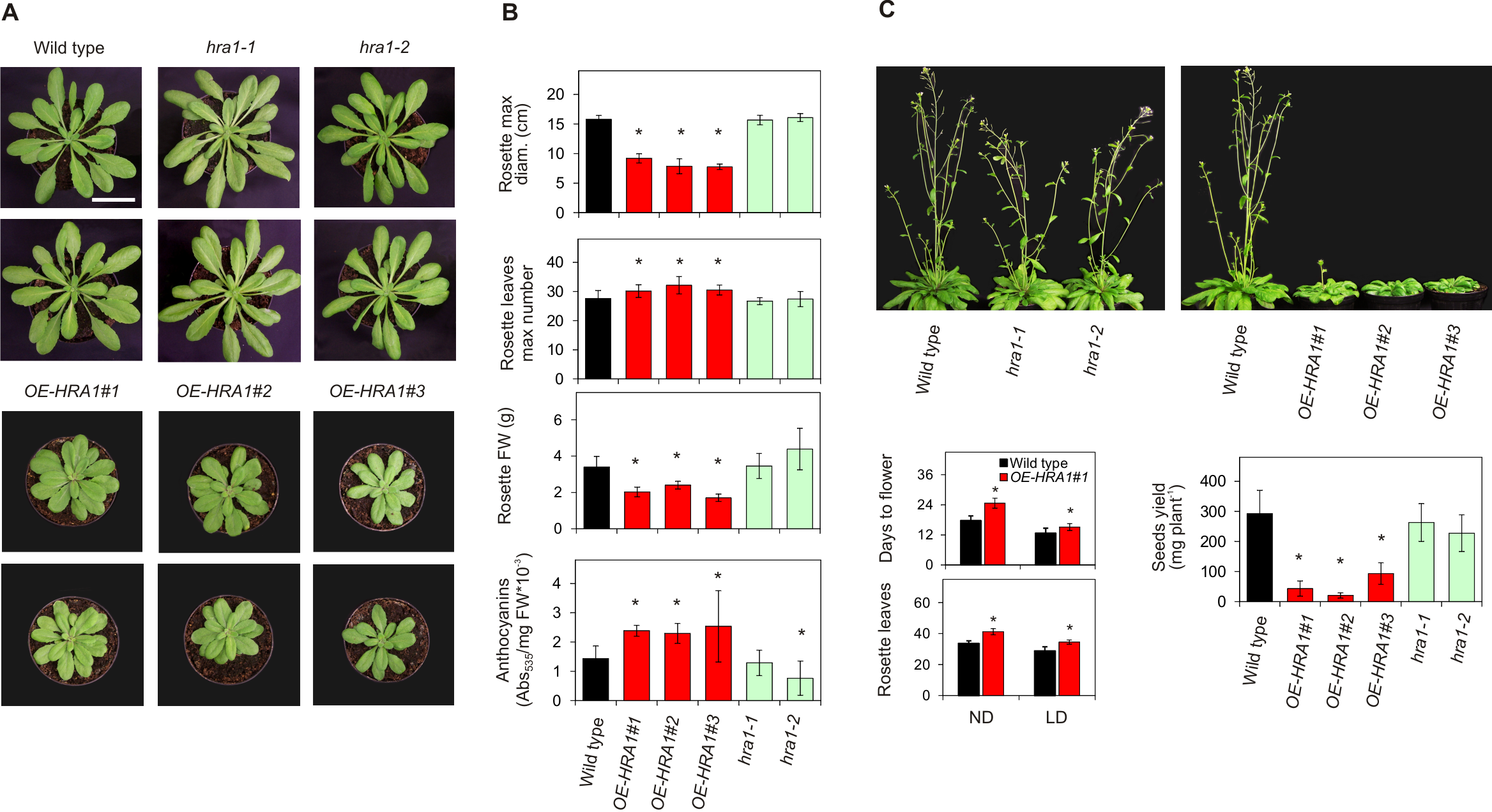

Supplement: Figure S13 — Phenotypical characterization of HRA1 overexpressing plants. (A) Representative pictures of wild type, HRA1 mutant, and overexpressing plants, grown at the end of the vegetative phase (stage 3.90 [30], here corresponding to 26 d of growth in soil under a 12 h light∶12 h darkness neutral photoperiod). Scale bar, 3 cm. OE-HRA1#1 and OE-HRA1#2 are 35:HRA1:FLAG overexpressors, and OE-HRA1#3 indicates, instead, an independent 35S:HRA1 transgenic line. Note the shortened petiole and reduced leaf index (ratio between length and width) in the overexpressing plants. No obvious differences from the wild type were recorded in the hra1-1 or hra1-2 mutants. (B) Rosette growth parameters and total leaf anthocyanin content in OE-HRA1 soil-grown plants at a stage corresponding to stage 3.90 in the wild type [30]. Overexpressing plants are more compact (reduced petiole elongation and lower rosette max diameter) and produce a higher number of leaves, which however are smaller than in the wild type (lower total fresh weight). Data are mean ± s.d. (n = 4 for rosette FW and anthocyanin content; n = 6 for rosette size and leaf number). *p<0.05 statistically significant means from the wild type, after one-way ANOVA. (C) Above, phenotype of plants progressing through the reproductive phase (principal stage 4 [30]; 46-d-old plants were photographed here). A developmental delay and partial loss of apical dominance was highlighted in the OE-HRA1 genotypes, whereas HRA1 mutants did not display significant alterations. Flowering time of OE-HRA1#1 plants (bottom left) was assessed both in terms of days from germination and number of leaves to floral induction and consistently showed a delay in the transgenic plants if compared with the wild type, independent of the photoperiodic flowering pathway (LD, long day, 15 h light∶9 h darkness; ND, neutral day). Mean ± s.d. (n = 12), *p<0.05 statistically significant means from the wild type, after one-way ANOVA. Data describing plant yield (bottom right [file pbio.1001950.s013.tif]

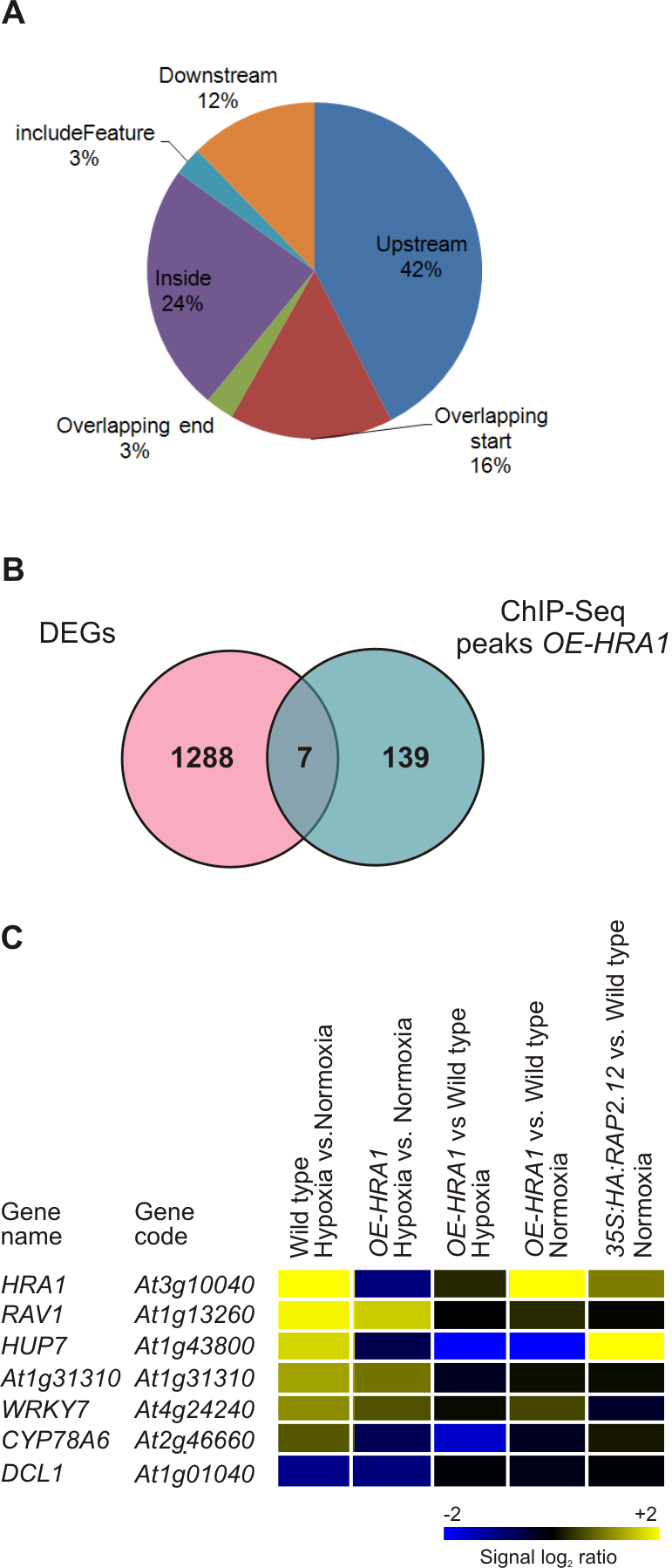

Supplement: Figure S14 — Identification of HRA1 binding sites by chromatin immunopurification and fragment sequencing (ChIP-seq). (A) Pie chart displaying the percentage of HRA1 binding peaks that map to defined domains of annotated genes. Upstream, peak maps 5′ of the predicted transcription start site; downstream, peak maps 3′ of the transcriptional unit of the gene; inside, peak maps within the annotated mRNA; overlapStart, peak overlaps with the predicted transcriptional start site of the gene; overlapEnd, peak overlaps with the end of the gene; includeFeature, peak includes the entire gene. (B) Venn diagram comparison of the 1,295 DEGs, identified by five comparisons in DNA microarray analysis, and the 146 putative targets of HRA1 binding, identified by ChIP-Seq. The overlap was seven genes. (C) Heatmap summarizing the expression values of the seven genes identified in both the DEG and ChIP-Seq datasets (Figure S14B), as extracted from the DNA microarray datasets. (TIF) [file pbio.1001950.s014.tif]

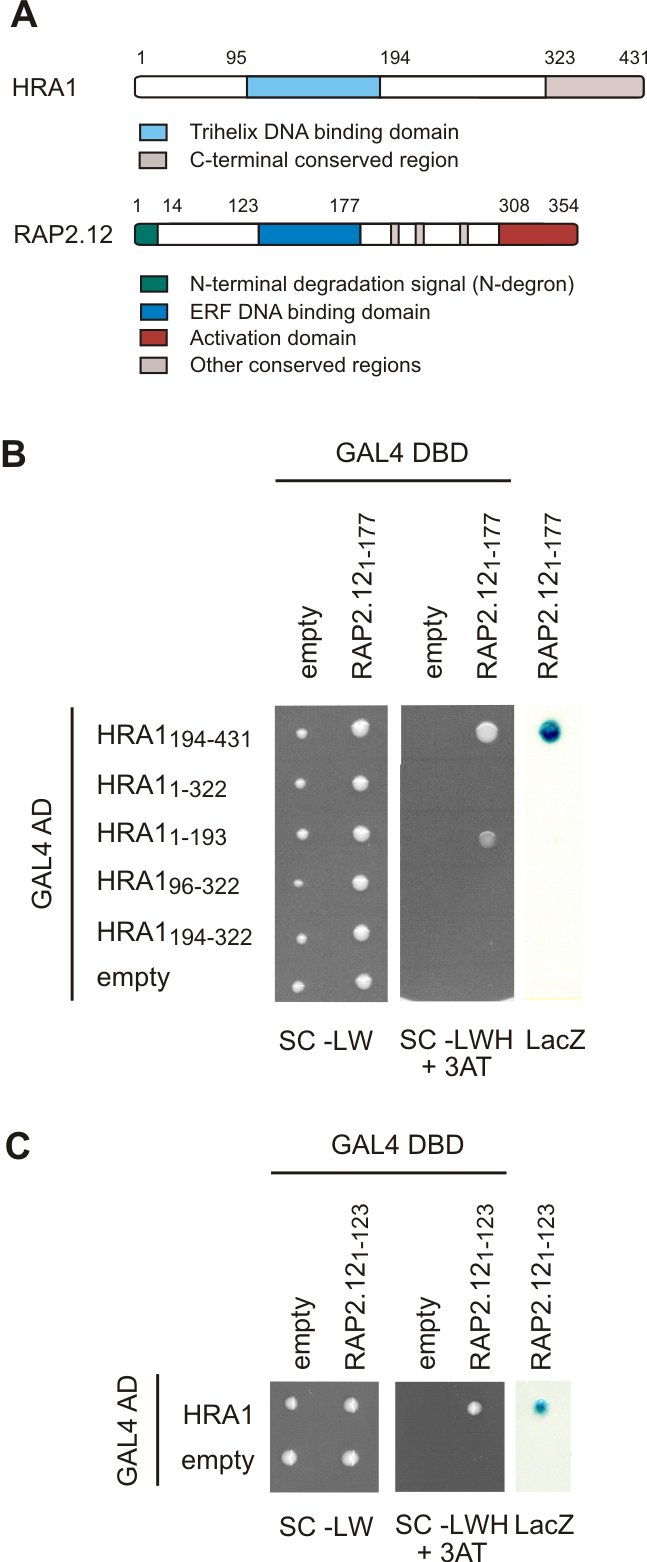

Supplement: Figure S15 — HRA1 interacts with RAP2.12. (A) Schematic view of the domains present on HRA1 and RAP2.12 proteins. (B) Yeast-two-hybrid between an N-terminal RAP2.12 fragment (bait) and HRA1 (prey), showing that amino acids 1–123 of RAP2.12, corresponding to a variable region preceding the ERF DNA binding domain [7], are sufficient for the interaction. (C) An N-terminal HRA1 deletion version (HRA1194–431), lacking the predicted trihelix DNA binding domain, is able to interact with RAP2.12 (RAP2.121–177) in the yeast-two-hybrid assay and also when used as the prey construct. (TIF) [file pbio.1001950.s015.tif]

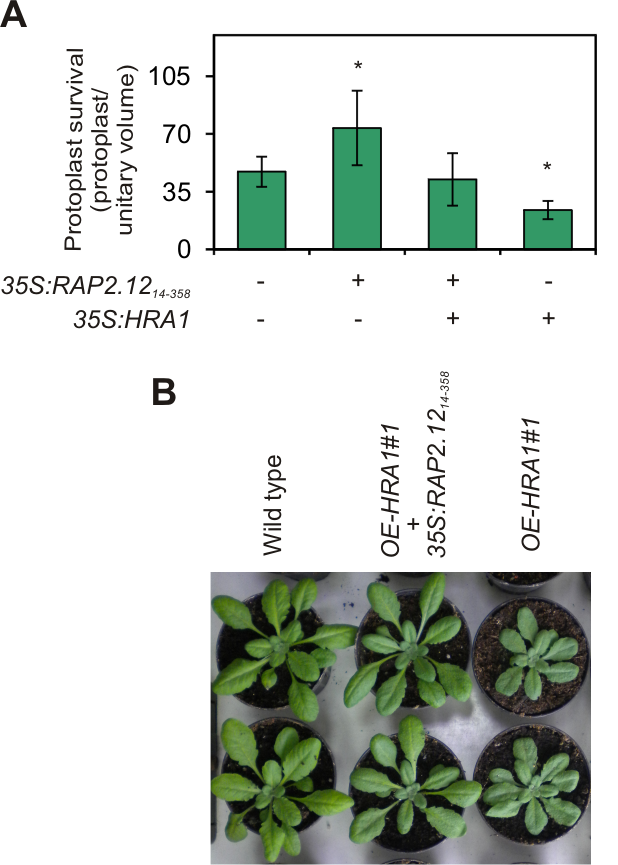

Supplement: Figure S16 — Interaction of HRA1 with RAP2.12 has an impact in vivo. (A) Survival of protoplasts incubated for 18 h was evaluated by calculating the proportion of alive, fluorescein diacetate-stained, cells [58] per unit volume on a hemocytometer under a fluorescence microscope. Incubation of protoplasts was static and in darkness, making the occurrence of hypoxia in the cells very likely. Data are mean ± s.d. (n = 8), and *p<0.05 indicates statistically significant difference from the control transformation (first column, protoplasts transformed with 35S:RrLuc only). Protoplasts were transfected with 3 µg individual plasmid DNA in every transformation. (B) Phenotype of soil-grown plants overexpressing HRA1 alone or HRA1 and the stable RAP2.1214–358 version at the same time. (TIF) [file pbio.1001950.s016.tif]
