# Supplementary material for: A Trihelix DNA Binding Protein Counterbalances Hypoxia-Responsive Transcriptional Activation in Arabidopsis
Source: PLoS Biol. 2014 Sep 16;12(9):e1001950. doi: 10.1371/journal.pbio.1001950 (PMC4165759; doi:10.1371/journal.pbio.1001950)
Supplement: Table S1 — List of low oxygen-inducible trihelix coding genes in four mono- and dicotyledonous plant species. In bold are genes belonging to the same orthologous group as HRA1 according to the PlantTFDB database. (DOCX) [file pbio.1001950.s017.docx]

| **Organism** | **Number genes** | **Gene IDs** | **References** |
| --- | --- | --- | --- |
| Arabidopsis | 1 | *At3g10040* | [1] |
| Rice | 1 | ***LOC_Os01g21590.1*** | [1] |
| Poplar | 4 | *pmrna11072, CX177654, pmrna37656,* ***pmrna35920*** | [2] |
| Soybean | 4 | *Glyma01g29760.1, Glyma06g15500.1,* ***Glyma13g21350.1, Glyma19g37410.1*** | [3] |

1. Kaplan-Levy RN, Brewer PB, Quon T, Smyth DR (2012) The trihelix family of transcription factors – light, stress and development. Trends Plant Sci 17: 163-171.
2. [Kreuzwieser J](http://www.ncbi.nlm.nih.gov/pubmed?term=Kreuzwieser%20J%5BAuthor%5D&cauthor=true&cauthor_uid=19005089), [Hauberg J](http://www.ncbi.nlm.nih.gov/pubmed?term=Hauberg%20J%5BAuthor%5D&cauthor=true&cauthor_uid=19005089), [Howell KA](http://www.ncbi.nlm.nih.gov/pubmed?term=Howell%20KA%5BAuthor%5D&cauthor=true&cauthor_uid=19005089), [Carroll A](http://www.ncbi.nlm.nih.gov/pubmed?term=Carroll%20A%5BAuthor%5D&cauthor=true&cauthor_uid=19005089), [Rennenberg H](http://www.ncbi.nlm.nih.gov/pubmed?term=Rennenberg%20H%5BAuthor%5D&cauthor=true&cauthor_uid=19005089), et al. (2009) Differential response of gray poplar leaves and roots underpins stress adaptation during hypoxia. Plant Physiol 149: 461-473.

1. [Nanjo Y](http://www.ncbi.nlm.nih.gov/pubmed?term=Nanjo%20Y%5BAuthor%5D&cauthor=true&cauthor_uid=21656040), [Maruyama K](http://www.ncbi.nlm.nih.gov/pubmed?term=Maruyama%20K%5BAuthor%5D&cauthor=true&cauthor_uid=21656040), [Yasue H](http://www.ncbi.nlm.nih.gov/pubmed?term=Yasue%20H%5BAuthor%5D&cauthor=true&cauthor_uid=21656040), [Yamaguchi-Shinozaki K](http://www.ncbi.nlm.nih.gov/pubmed?term=Yamaguchi-Shinozaki%20K%5BAuthor%5D&cauthor=true&cauthor_uid=21656040), [Shinozaki K](http://www.ncbi.nlm.nih.gov/pubmed?term=Shinozaki%20K%5BAuthor%5D&cauthor=true&cauthor_uid=21656040), et al. (2011) Transcriptional responses to flooding stress in roots including hypocotyl of soybean seedlings. Plant Mol Biol 77: 129-144.
