# Supplementary material for: A Trihelix DNA Binding Protein Counterbalances Hypoxia-Responsive Transcriptional Activation in Arabidopsis
Source: PLoS Biol. 2014 Sep 16;12(9):e1001950. doi: 10.1371/journal.pbio.1001950 (PMC4165759; doi:10.1371/journal.pbio.1001950)
Supplement: Table S4 — List of ChIP-PCR primers. (DOCX) [file pbio.1001950.s020.docx]

| **Primer name** | **Primer sequence (5’-3’)** |
| --- | --- |
| PrimersetI_Fw | GATGAGCGTTTTAGCCACTG |
| PrimersetI_Rv | GGCTAGCCTCAAATTGCTTG |
| PrimersetII_Fw | CAAGCAATTTGAGGCTAGCC |
| PrimersetII_Rv | TCAAGAGCAATATCAAGAGCATC |
| PrimersetIII_Fw | GATGCTCTTGATATTGCTCTTGA |
| PrimersetIII_Rv | AGCTAGATACAGGGACAAACA |
| PrimersetIV_Fw | CGAGAAAAAGTGTTTGTCCCTGT |
| PrimersetIV_Rv | TGCCAGCCTTAAACTGAGAGT |
| PrimersetV_Fw | AGTCACAATCATTACGCGGT |
| PrimersetV_Rv | ACTGTTTTTACCTTTCTGCCA |
| PrimersetVI_Fw | AACCAGCCCCTTGGTTTAGGC |
| PrimersetVI_Rv | CAGTGGTTTTGGGAGCCGTCA |
