# Supplementary material for: A Trihelix DNA Binding Protein Counterbalances Hypoxia-Responsive Transcriptional Activation in Arabidopsis
Source: PLoS Biol. 2014 Sep 16;12(9):e1001950. doi: 10.1371/journal.pbio.1001950 (PMC4165759; doi:10.1371/journal.pbio.1001950)
Supplement: Table S5 — Comparison of total HRA1, endogenous HRA1 and ADH1 transcript levels in wild type, and OE-HRA1 and hra1 seedlings in normoxia or after 2 h hypoxia. Fold change values were calculated relatively to the wild type control. Data are means ± s.d. (n = 4). *p adj. <0.05 and **p adj. <0.01 were calculated separately for control and hypoxic conditions, after one-way-ANOVA. (DOCX) [file pbio.1001950.s021.docx]

| **Control** |  |  |  |  |  |  |  |  |  |  |  |  |  |  |
| --- | --- | --- | --- | --- | --- | --- | --- | --- | --- | --- | --- | --- | --- | --- |
|  | **Wild type** |  | ***OE-HRA1#1*** |  |  | ***OE-HRA1#2*** |  |  | ***hra1-1*** |  |  | ***hra1-2*** |  |  |
| **mRNA** | **Mean value** | **s.d.** | **Mean value** | **s.d.** | **p adj** | **Mean value** | **s.d.** | **p adj** | **Mean value** | **s.d.** | **p adj** | **Mean value** | **s.d.** | **p adj** |
| ***HRA1_Tot_*** | 0.96 | 0.05 | 156.74 | 14.91 | ** | 167.61 | 10.57 | ** | 1.81 | 0.24 |  | 3.48 | 0.49 |  |
| ***HRA1_Endo_*** | 0.94 | 0.08 | 0.14 | 0.01 | ** | 0.12 | 0.02 | ** | 0.00 | 0.00 | ** | 0.00 | 0.00 | ** |
| ***ADH1*** | 1.04 | 0.06 | 0.68 | 0.01 | ** | 0.63 | 0.02 | ** | 1.29 | 0.11 | * | 1.33 | 0.09 | * |
| **Hypoxia** |  |  |  |  |  |  |  |  |  |  |  |  |  |  |
|  | **Wild type** |  | ***OE-HRA1#1*** |  |  | ***OE-HRA1#2*** |  |  | ***hra1-1*** |  |  | ***hra1-2*** |  |  |
| **mRNA** | **Mean value** | **s.d.** | **Mean value** | **s.d.** | **p adj** | **Mean value** | **s.d.** | **p adj** | **Mean value** | **s.d.** | **p adj** | **Mean value** | **s.d.** | **p adj** |
| ***HRA1_Tot_*** | 41.98 | 4.12 | 121.21 | 17.30 | ** | 122.79 | 3.87 | ** | 38.92 | 0.38 |  | 59.39 | 27.17 |  |
| ***HRA1_Endo_*** | 59.07 | 7.88 | 0.95 | 0.14 | ** | 1.50 | 0.11 | ** | 0.06 | 0.08 | ** | 0.06 | 0.08 | ** |
| ***ADH1*** | 63.35 | 0.67 | 5.80 | 0.13 | ** | 6.33 | 0.62 | ** | 76.28 | 9.50 | * | 72.53 | 6.53 |  |
