# Supplementary material for: A Trihelix DNA Binding Protein Counterbalances Hypoxia-Responsive Transcriptional Activation in Arabidopsis
Source: PLoS Biol. 2014 Sep 16;12(9):e1001950. doi: 10.1371/journal.pbio.1001950 (PMC4165759; doi:10.1371/journal.pbio.1001950)
Supplement: Table S6 — Referenced list of the plasmid constructs produced in this study. (DOCX) [file pbio.1001950.s022.docx]

| **Construct name** | **Cloning vector** | **Reference** |
| --- | --- | --- |
| 35S-HRA1-FLAG | p35S:GATA-HF | [1] |
| 35S-HRA1 | pK7WG2 | [2] |
| 35S-HRA1-GFP | p35S:GATA-HF | [1] |
| promHRA1:GUS | PKGWFS7 | [2] |
| promHRA1:PpLuc | pGWL7 | This study |
| promPDC1:PpLuc | pGWL7 | This study |
| 35S:PpLuc | P2GW7 | [2] |
| 35S:HRA1 | p2GW7 | [2] |
| 35S:HRA1_194-431_ | p2GW7 | [2] |
| 35S:RAP2.12_14-358_ | p2GW7 | [2] |
| 35S:HRA1:YFPn | pDH51-GW-YFPn | [3] |
| 35S:RAP2.12_14-358_:YFPc | pDH51-GW-YFPc | [3] |
| GAL4 DBD:RAP2.12_1-123_ | pDEST^TM^32 | ProQuest^TM^ Two-Hybrid (Life Technologies) |
| GAL4 DBD: RAP2.12_1-177_ | pDEST^TM^32 | ProQuest^TM^ Two-Hybrid (Life Technologies) |
| GAL4 AD:HRA1 | pDEST^TM^22 | ProQuest^TM^ Two-Hybrid (Life Technologies) |
| GAL4 AD: HRA1_194-431_ | pDEST^TM^22 | ProQuest^TM^ Two-Hybrid (Life Technologies) |

1. Mustroph A, Lee SC, Oosumi T, Zanetti ME, Yang H, ET AL. (2010) Cross-kingdom comparison of transcriptomic adjustments to low-oxygen stress highlights conserved and plant-specific responses. Plant Physiol 152: 1484-1500.
2. Karimi M, Inzé D, Depicker A (2002) GATEWAY™ vectors for Agrobacterium-mediated plant transformation. Trends Plant Sci 7: 193-195.
3. Zhong S, Lin Z, Fray R, Grierson D (2008) Improved plant transformation vectors for fluorescent protein tagging. Transgenic Res 17: 985-989.
