# Supplementary material for: A Trihelix DNA Binding Protein Counterbalances Hypoxia-Responsive Transcriptional Activation in Arabidopsis
Source: PLoS Biol. 2014 Sep 16;12(9):e1001950. doi: 10.1371/journal.pbio.1001950 (PMC4165759; doi:10.1371/journal.pbio.1001950)
Supplement: Table S7 — Oligonucleotide primers used for gene cloning and RT-PCR screening. (DOCX) [file pbio.1001950.s023.docx]

| **Primer name** | **Primer sequence (5’-3’)** |
| --- | --- |
| Primerset1_Fw | gaactcgaagatactgtac |
| Primerset1_Rv | tgcggcatttctaagctc |
| Primerset2_Fw | cggcaagtgtagtggaagat |
| Primerset2_Rv | cccttagcggaactcgggtca |
| gwHRA1_Fw | caccatggaatcgaatgtgatgtt |
| gwHRA1_580_Fw | caccatgggtaaaggaatcgcttgtc |
| gwHRA1_Rv | tcaccccttagcggaa |
| gwHRA1_5’UTR_Fw | caccatggaatcgaatgtgatgttctctggg |
| gwHRA1_3’UTR_Rv | ccccttagcggaactcgggtcaac |
| gwpromHRA1_Fw | caccatatgatgcttaattatatgatgg |
| gwpromHRA1_Rv | caaacactgaaccctaaattggat |
| gwpromPDC1_Fw | cacccttgaattacacta |
| gwpromPDC1_Rv | ggaaatgggaaggtttgagtt |
| LBb1 | gcgtggaccgcttgctgcaact |
|  |  |
