# Supplementary material for: A Trihelix DNA Binding Protein Counterbalances Hypoxia-Responsive Transcriptional Activation in Arabidopsis
Source: PLoS Biol. 2014 Sep 16;12(9):e1001950. doi: 10.1371/journal.pbio.1001950 (PMC4165759; doi:10.1371/journal.pbio.1001950)
Supplement: Table S8 — Oligonucleotide primers used for qPCR analyses. (DOCX) [file pbio.1001950.s024.docx]

| **Locus name and AGI code** | **Primer name** | **Primer sequence (5’-3’)** |
| --- | --- | --- |
| *Ubiquitin10 (At4g05320)* | sgUBQ10_Fw | ggccttgtataatccctgatgaataag |
|  | sgUBQ10_Rv | aaagagataacaggaacggaaacatagt |
| *Tubulin (At5g62690)* | sqTUB2_Fw | acaccagacatagtagcagaaatcaag |
|  | sqTUB2_Rv | actcgttgggaggaggaact |
| *HRA1 (At3g10040)* | sgHRA1_Fw | tcatgttacggcggagtgaa |
|  | sgHRA1_Rv | caacccgtgtacccgaagac |
|  | sgHRA1_Endo_Fw | gggaagaagcggcaagtgtagtg |
|  | sgHRA1_Endo_Rv | tttactgcctaatgtcactaaaacgtgag |
|  | sgHRA1_Tot_Fw | agtcagcagcagaactgttttcacg |
|  | sgHRA1_Tot_Rv | tctccactccttcccactcataccc |
| *RAP2.12 (At1g53910)* | sgRAP2.12_Fw | actgaatgggacgcttcactgg |
|  | sgRAP2.12_Rv | agggtttgcaccattgtcctgag |
| *ADH1 (At1g77120)* | sgADH1_Fw | tattcgatgcaaagctgctgtg |
|  | SgADH1_Rv | cgaacttcgtgtttctgcggt |
| *HB1 (At2g16060)* | sgHb1_Fw | tttgaggtggccaagtatgca |
|  | sgHb1_Rv | tgatcataagcctgaccccaa |
| *PDC1 (At4g33070)* | sgPDC1_Fw | cgattatggcactaaccggatt |
|  | sgPDC_1Rv | tgttcaccaccgcctgataac |
| *HUP43 (At5g39890)* | sgHUP43_Fw | cttcgagccgttttggatga |
|  | sgHUP43_Rv | acgtcactaacggagatcgtcc |
| *LBD41 (At3g02550)* | sgLBD41_Fw | tgaagcgcaagctaacgca |
|  | sgLBD41_Rv | atcccaggacgaaggtgattg |
| *HUP7 (At1g43800)* | sgHUP7_Fw | accaatgttggcaacccgcttc |
|  | sgHUP7_Rv | tttccctcagctcacgaacctg |
|  |  |  |
